# A multi-decadal record of oceanographic changes of the past ~165 years (1850-2015 AD) from Northwest of Iceland

**DOI:** 10.1371/journal.pone.0239373

**Published:** 2020-09-29

**Authors:** Margit H. Simon, Francesco Muschitiello, Amandine A. Tisserand, Are Olsen, Matthias Moros, Kerstin Perner, Siv Tone Bårdsnes, Trond M. Dokken, Eystein Jansen

**Affiliations:** 1 NORCE Norwegian Research Centre, Bjerknes Centre for Climate Research, Bergen, Norway; 2 Department of Geography, Cambridge University, Cambridge, United Kingdom; 3 Geophysical Institute, Bjerknes Centre for Climate Research, University of Bergen, Bergen, Norway; 4 Department of Marine Geology, Leibniz Institute for Baltic Sea Research, Rostock, Germany; 5 Department of Earth Sciences, Bjerknes Centre for Climate Research, University of Bergen, Bergen, Norway; Aarhus Universitet, DENMARK

## Abstract

Extending oceanographic data beyond the instrumental period is highly needed to better characterize and understand multi-decadal to centennial natural ocean variability. Here, a stable isotope record at unprecedented temporal resolution (1 to 2 years) from a new marine core retrieved off western North Iceland is presented. We aim to better constrain the variability of subsurface, Atlantic-derived Subpolar Mode Water (SPMW), using near surface-dwelling planktic foraminifera and Arctic Intermediate Water (AIW) mass changes using benthic foraminifera over the last ~165 years. The reconstruction overlaps in time with instrumental observations and a direct comparison reveals that the δ^18^O record of *Neogloboquadrina pachyderma* is reliably representing temperature fluctuations in the SPMWs. Trends in the *N*. *pachyderma* δ^13^C record match the measured phosphate concentration in the upper 200 m on the North Icelandic Shelf well. Near surface-dwelling foraminifera trace anthropogenic CO_2_ in the Iceland Sea by ~ 1950 **±** 8, however, a reduced amplitude shift in the Marine Suess effect is identified. We argue that this is caused by a contemporary ongoing increase in marine primary productivity in the upper ocean due to enhanced Greenland’s freshwater discharge that has contributed to a nutrient-driven fertilization since the 1940s/50s (Perner et al., 2019). Multi-decadal variability is detected. We find that the 16-year periodicity evident in SPMW and AIWs based on the δ^18^O of *N*. *pachyderma* and *M*. *barleeanum* is a signal of SST anomalies propagated into the Nordic Seas via the Atlantic inflow branches around Iceland. Spectral analyses of the planktic foraminiferal δ^13^C signal indicate intermittent 30-year cycles that are likely reflecting the ocean response to atmospheric variability, presumably the East Atlantic Pattern. A long-term trend in benthic δ^18^O suggests that Atlantic-derived waters are expanding their core within the water column from the subsurface into deeper intermediate depths towards the present day. This is a result of increased transport by the North Icelandic Irminger Current to the North Iceland Shelf over the historical era.

## 1. Introduction

A key problem for reducing the uncertainty in future climate projections is that historical records are too short to test the skill of climate models, raising concerns on our ability to successfully project future change for any given emission scenario. In particular, the lack of records with a high temporal resolution before the 1950s limits our understanding of the interplay among the ocean, atmosphere, and cryosphere, and the impact of anthropogenic forcing upon regional climate on multi-decadal and shorter timescales.

Various studies have used observations and model simulations to identify patterns of natural ocean variability in the North Atlantic on decadal to century time-scales [1, e.g. 2] but there is still a need for studies resolving annual to multi-decadal climate variability beyond the instrumental observations. Especially in the Northern Seas, which includes the northern North Atlantic, the Nordic Seas, and the Arctic Ocean [3], where modes such as the North Atlantic Oscillation (NAO) [4], and Atlantic Multidecadal Oscillation (AMO) are among the ones that have the strongest impact on observed climate variability [5]. An ongoing question in the community is, however, if the detected climate variability is an internal mode of the climate system or is externally forced [6–9]. Evidence for multidecadal variability in records over the last 1 ka indicate that this type of variability may be internal (for example, ocean heat storage and transport), or alternatively linked to the interactions between fluctuations in total solar irradiance and volcanic aerosols [10–13].

Here, we present a new marine sediment record providing a high-resolution perspective on ocean climate variability from the western North Icelandic shelf (NIS), a region that is sensitive to broad-scale climatic and oceanographic changes in the North Atlantic [14].

**Fig 1:**
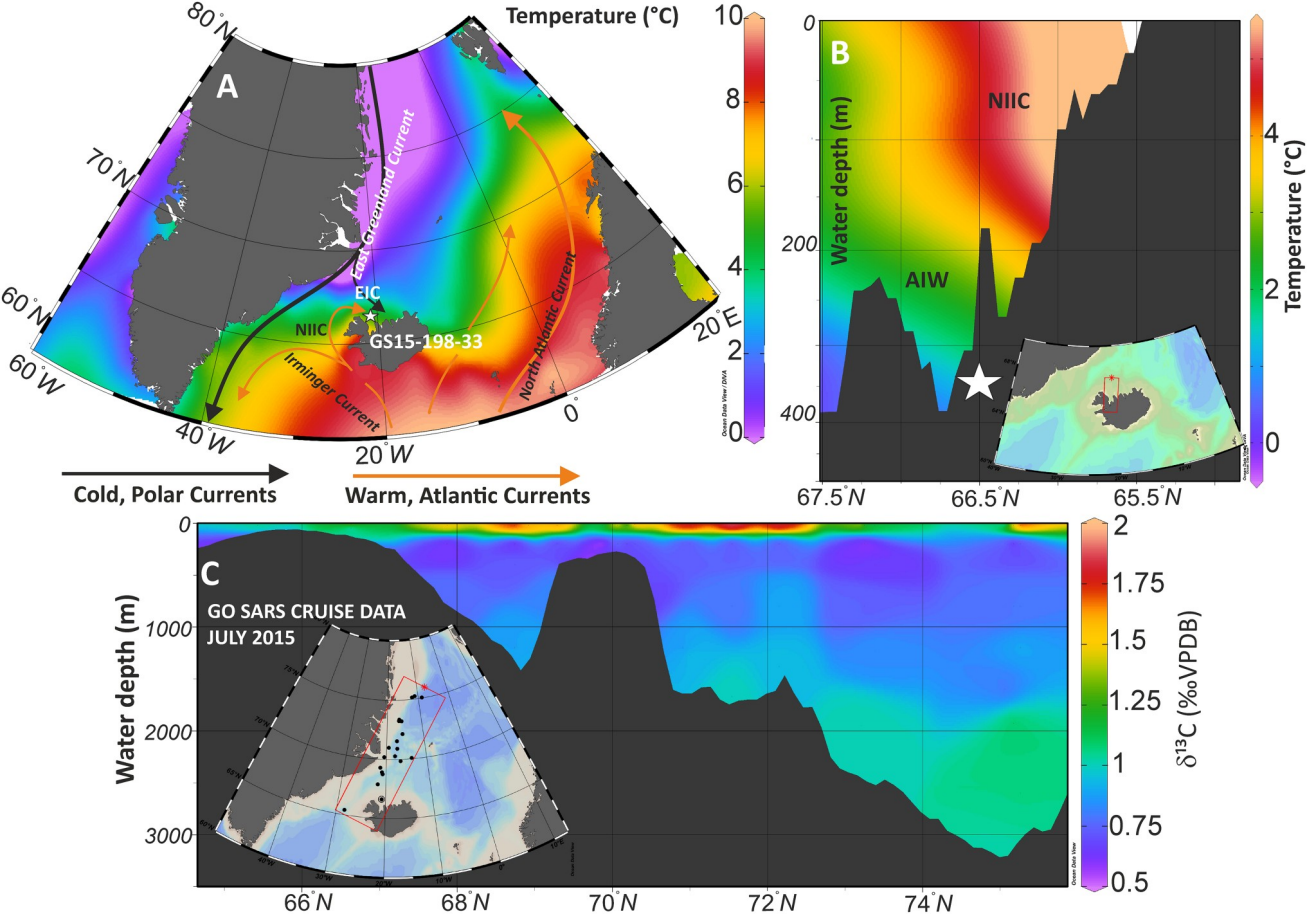
Modern oceanography and core location. (A) Summer surface temperatures (World Ocean Atlas 2018 (WOA 18), April-September) in the northern North Atlantic. The white star indicates the core location of GS15-198-33 on the northwestern Icelandic Shelf. The location of the major ocean currents is indicated with the arrows. (B) North to South hydrographic section of modern summer temperatures [19] across the core location showing warmer NIIC waters overlying cold Arctic/Polar intermediate waters (AIW) in the deep troughs. EIC: East Iceland Current; NIIC: North Iceland Irminger Current. (C) Cross-section of stable isotopes of dissolved inorganic carbon (δ^13^C of DIC in ‰ VPDB) in the seawater samples analysed over the entire water column collected during ice2ice I cruise in July 2015. The sampling locations are shown in the insert. Ocean data view was used for creating the maps [20].

In the region offshore North Iceland surface and subsurface water conditions are sensitive to the varying influence of the Polar-sourced East Icelandic Current (EIC) and the Atlantic-sourced North Icelandic Irminger Current (NIIC) (Fig 1). The NIIC is one of three main branches of Atlantic Water inflow to the Nordic Seas [3]. The relative contribution of these currents to the shelf area is closely related to atmospheric forcing. In particular, the Atlantic Water contribution to the North Icelandic realm strengthens during periods dominated by southeasterly winds [15, 16]. The NIIC is the weakest of the three branches of the Atlantic Water inflow to the Nordic Seas in terms of volume transport. It provides only some 10% of the Atlantic Water and 8% of the heat transport to the Nordic Seas [17]. However, it has been suggested that it is important for the formation of the North Icelandic Jet, which is a substantial component of the Denmark Strait overflow, and may therefore contribute to the Atlantic and global thermohaline circulation [14]. It is also of fundamental importance for climate and the marine ecosystems around Iceland [18], by giving rise to a milder climate compared to other areas at the same latitude.

The new record presented in this study is unique as marine sediment archives that overlap with the instrumental period at such high temporal resolution are rare. Thus, it offers the opportunity to compare the geochemical measurements based on planktic and benthic foraminifera against available hydrographic data and extend the instrumental oceanographic records from the 1950s back to 1850 AD. The importance of such an an approach was recently demonstrated by Perner, Moros [21] providing a marine perspective on Greenland’s freshwater discharge to the ocean since 1850 from the same core location. The reconstructions suggest that the recent acceleration in freshwater releases from melting Arctic Ocean drift/sea ice and the eastern Greenland Ice Sheet entrained into the EGC started in the 1940s/50s and is unprecedented since 1850. This freshwater delivers high amounts of nutrients (dissolved organic carbon, P, Fe, and N) [22]. Thus, its input contributes to a nutrient-driven fertilization of the upper ocean and consequently increases the marine primary productivity since the 1940s/50s. The purpose of this paper is to reconstruct the properties of the subsurface and intermediate water for the past ~165 years. The goal is to get a better constrain the flow of Atlantic and Arctic Intermediate Water (AIW) on the western NIS in relation to climate over historical times on centennial to decadal time scales. Variability in the degree of AIW entrainment into the NIIC and therefore the SPMW/AIW composition of the waters influencing the NIS has previously been linked to changes Atlantic circulation dynamics such as variations in the strength of the Atlantic Meridional Overturning Circulation [23–25]. The new data presented here complements and extends the reconstructions of surface layer hydrology from Perner, Moros [21] to the deeper layers of the area.

A prominent feature of the historical period is the Marine Suess effect [26]. Fossil fuel CO_2_ has a δ^13^C value of -28‰ (VPDB) [27] and the penetration of this isotopically depleted signal into the oceans results in an overall decreasing δ^13^C signature of the surface ocean. The here presented record offers the opportunity to constrain the Suess effect impact on the foraminiferal carbon isotope signature in high detail for the Iceland Sea.

**Fig 2:**
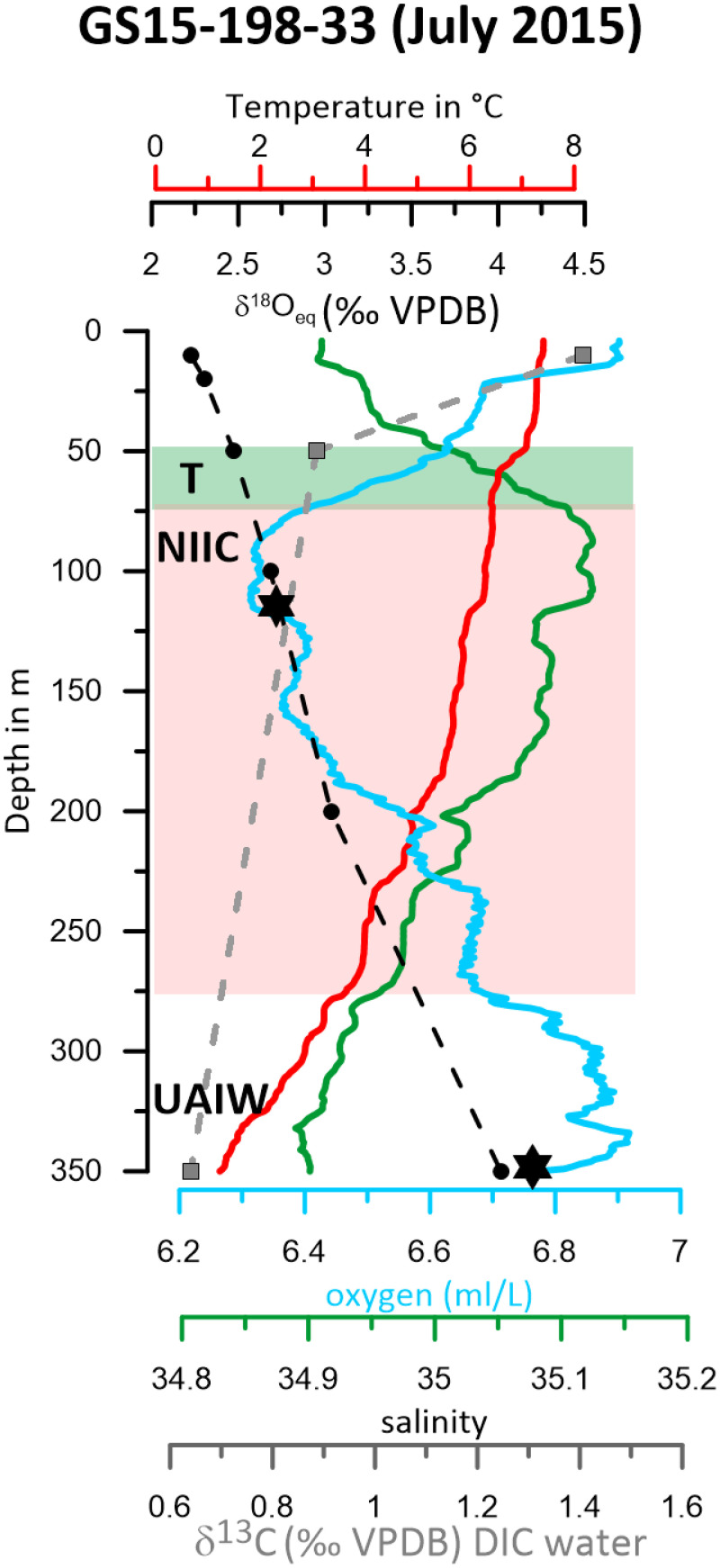
Water mass properties at the core location on the North Iceland shelf. Measured temperature, salinity, and oxygen profiles from July 2015 during research cruise GS15-198. Black stars denote apparent calcification depth (the depth where δ^18^O_calcite_ matches δ^18^O_eq_) of planktic and benthic foraminifera. The calculated δ^18^O_eq_ in the water column is based on the measured temperature and δ^18^O of the water from the CTD data; (black circles). There is an offset between the δ^18^O_calcite_ and δ^18^O_eq_ for benthic species *M*. *barleeanum*. The stable isotope data for dissolved inorganic carbon (δ^13^C of DIC, δ^13^C_DIC_) in the seawater is shown as grey squares. The extent of Atlantic Water (>34.9 and >4°C) of the NIIC is outlined in the red box. Note that the Atlantic Water occupies intermediate depths in the water column, from approximately 75–275 m. Thermocline waters (T) are indicated between 50-75m as a green box. The surface is occupied by a fresh, relatively warm water mass. The bottom of the water column 275–350 m is dominated by colder and fresher Upper Arctic intermediate waters (UAIW).

**Fig 3:**
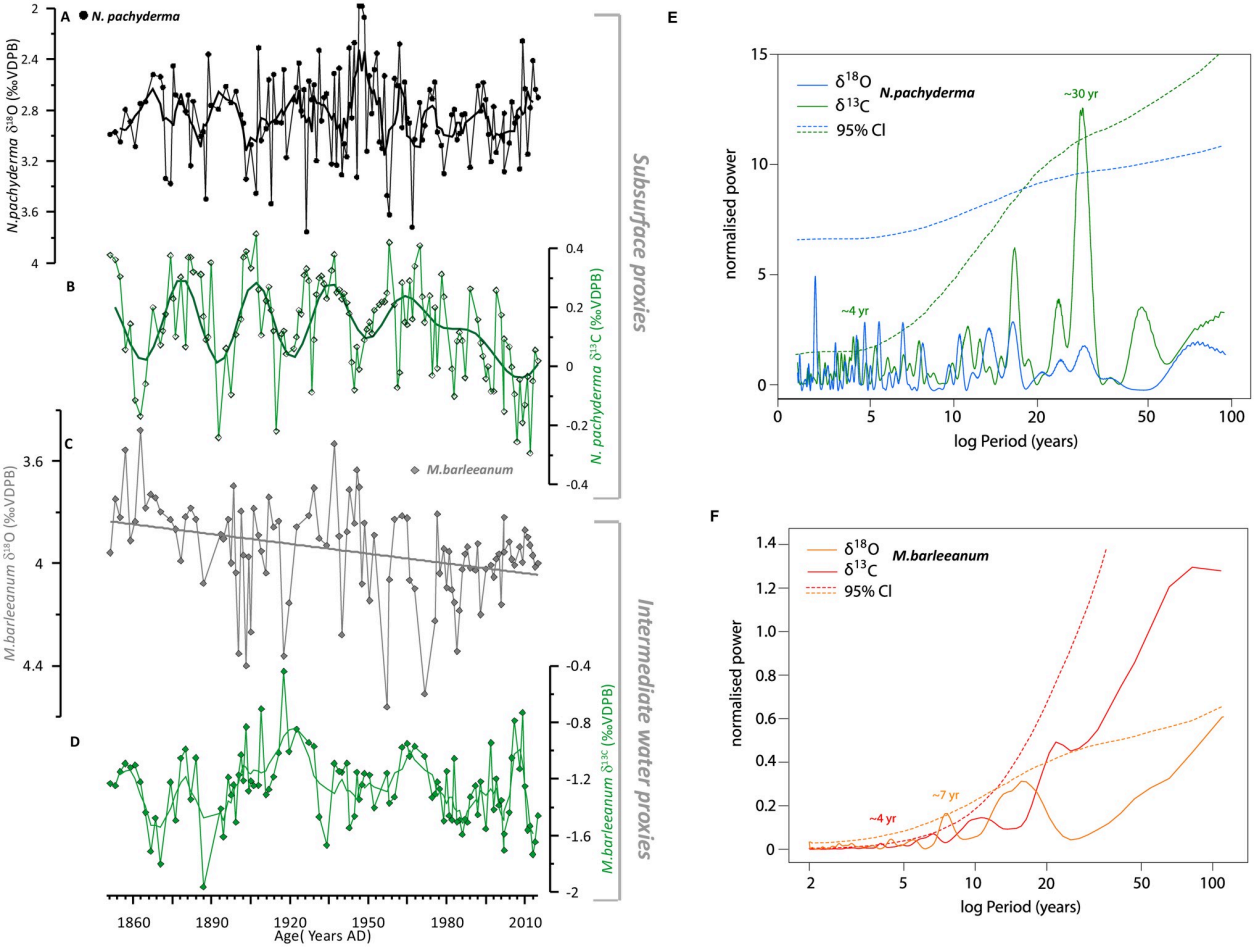
GS15-198 33 marine sediment core climate proxies over the historical time interval. (A) δ^18^O time series of *N*. *pachyderma* in black and 5 point running mean (thick line) (B) δ^13^C time series of *N*. *pachyderma* in green (raw data) plotted on top the 25-yrs high-pass filter to highlight the significant ~30 cycle identified in the frequency analysis. (C) δ^18^O time series of *M*. *barleeanum* (vital effect corrected) in grey, with the long-term trend highlighted as black linear regression (D) δ^13^C time series of *M*. *barleeanum* in green (raw data) and 5 point running mean as a thick line. Power spectral density functions of isotopic records for E) *N*.*pachyderma* and F) *M*.*barleeanum* from core GS15-198-33 using the Lomb-Scargle Fourier transform [57]. The spectra of the data are tested against the theoretical red-noise spectrum computed from simulated 20,000 AR1 series. The dashed curves are the estimated red-noise 95% confidence limits for the respective records. Significant periodicities (95% Cl) are also noted.

**Fig 4:**
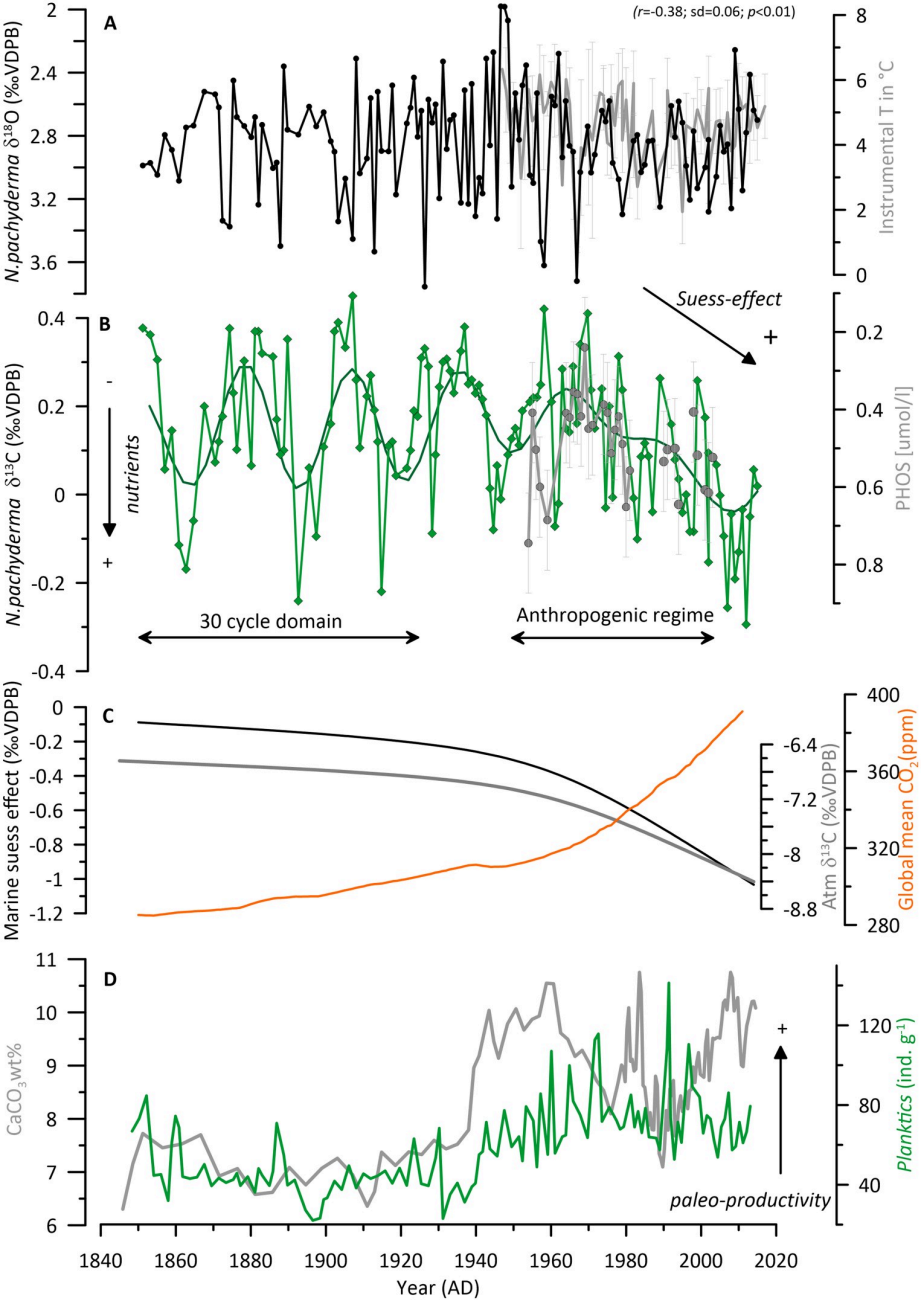
Comparison of proxies against instrumental observations and evaluation of the Marine Suess effect at core site GS15-198 33. (A) δ^18^O time series of *N*. *pachyderma* in black against instrumental temperature measurements (1SD) from Hunafloi station in grey (HF 3: 66.5 N; 20.78 W; S1 Fig in S1 File). Continuous instrumental data for that station was only available after 1974, hence we use data from the Siglunes section, situated further east and operated since 1947 (SI3: 66.32 N; 18.50 W; S1 Fig in S1 File) for the comparison in the older part of the record; coefficient of correlations *r* and 2 standard deviations are shown for reference. (B) δ^13^C time series of *N*.*pachyderma* in comparison with measured phosphate concentration (μmol kg ^−1^) in grey in the upper 200m on the NIS (68–66 °N, 22–18 °W; S2 Fig in S1 File) data retrieved from the International Council for the Exploration of the Sea (ICES) database: https://ocean.ices.dk/https://ocean.ices.dk in a narrow grid cell; δ^13^C of *N*.*pachyderma* is shown with the 25-yrs high-pass filter on top to highlight the significant ~30 cycle identified in the frequency analysis (Fig 3F). Indicated are the time intervals of significant multi-decadal frequency compared to before 1950´s and after when the Marine Suess effect dominates the regime. (C) estimated Marine Suess effect on the NIS at 100 m water depth in black; atmospheric δ^13^C record from Rubino, Etheridge [65] in grey; atmospheric CO_2_ concentration obtained from https://data.giss.nasa.gov/modelforce/ghgases/Fig1A.ext.txt in orange. (D) calcium carbonate content (CaCO_3_) of GS15-198 33 in green; the flux of planktic foraminifera g^-1^ in grey. Data from Perner, Moros [21] based on the same core material.

**Fig 5:**
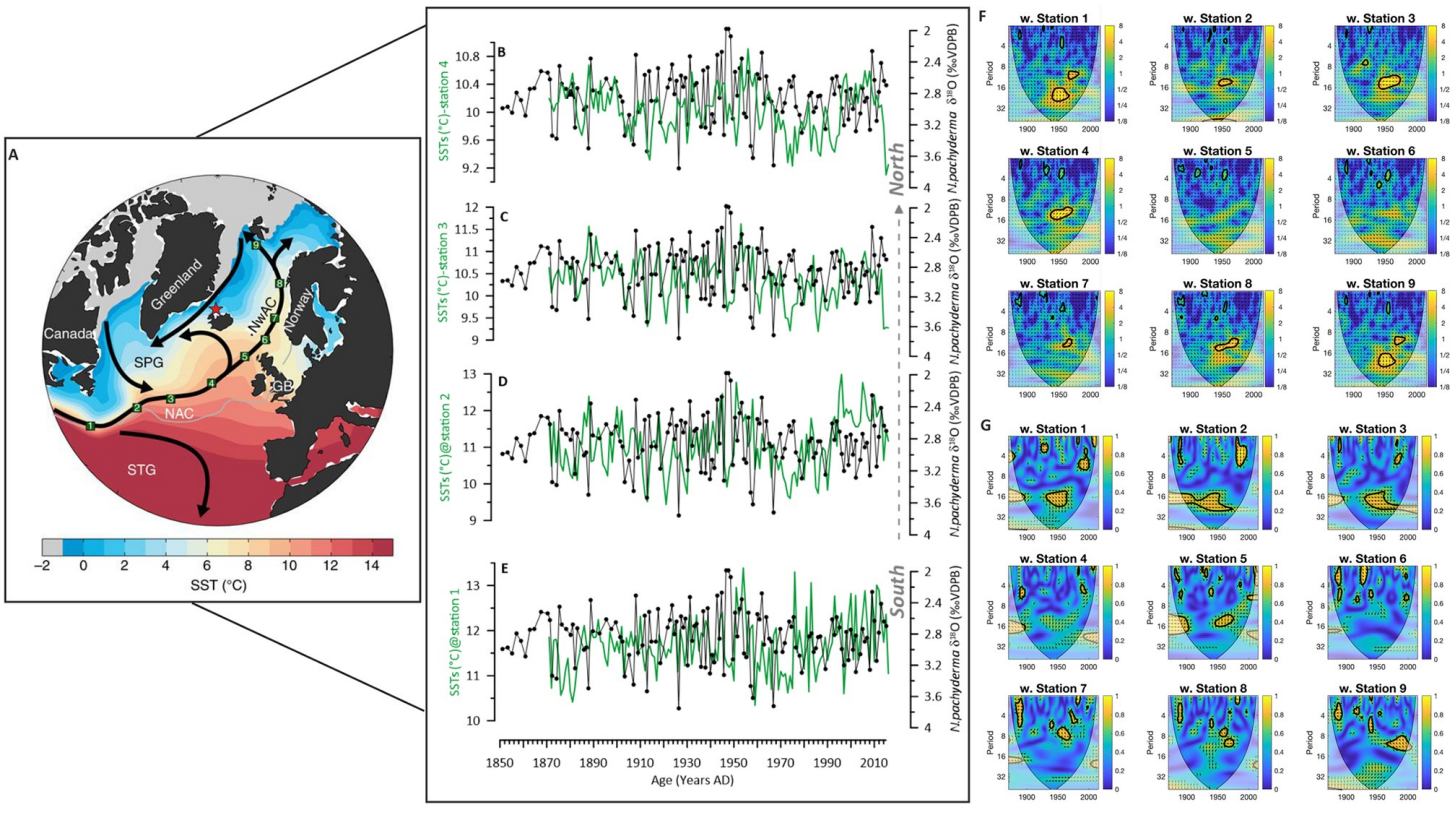
Poleward propagation of SST anomalies in the Nordic realm. (A) Map is taken from Årthun, Eldevik [81] with climatological SST (colour), major ocean surface currents (black arrows), and core location of marine sediment core GS15-198 33 of this study (red star). Sea ice is indicated by the grey shading. The green squares represent the selected stations (St1–9, e.g. St1 is the southernmost and st9 is the northernmost) along the NAC-NwAC pathway. The boundary between the subtropical gyre (STG) and subpolar gyre (SPG) is indicated by the time-mean zero SSH contour (grey line). (B-E) δ^18^O time series of *N*. *pachyderma* in black in comparison to data sets of Hadley Centre SST (HadISST), (°C) at st1-4 (in green).) (F) Cross Wavelet Transform (XWT) analysis between δ^18^O time series of *N*. *pachyderma* and all 9 stations as displayed in (A). (G) Wavelet coherence analysis (WTC) between the two time series for all stations. The thick black contour designates the 5% significance level against red noise and the cone of influence where edge effects might distort the picture is shown as a lighter shade.

**Fig 6:**
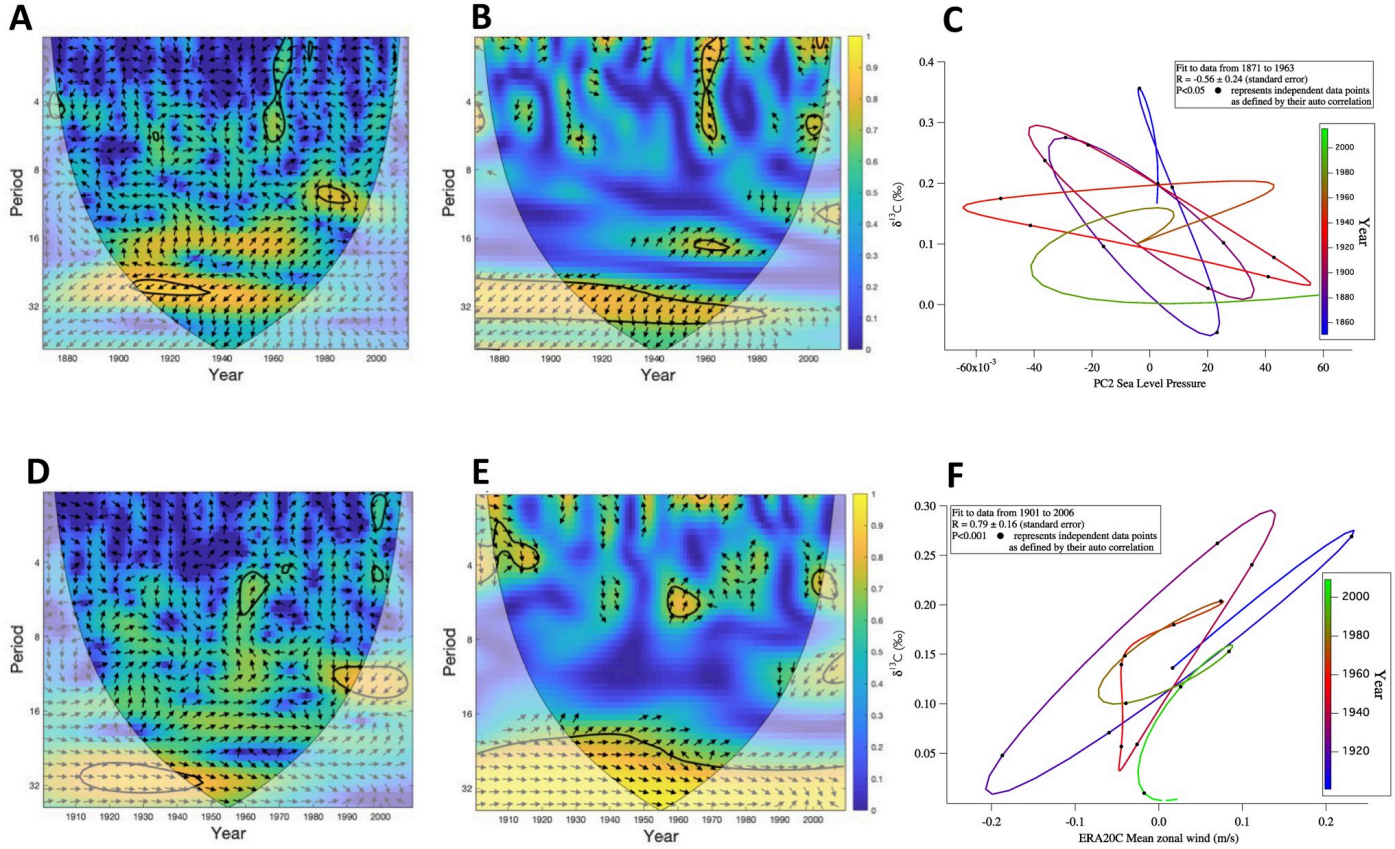
Comparison of stable carbon isotope time series with East Atlantic Pattern (EAP) and local wind forcing on the NIS. (A) Cross Wavelet Transform (XWT) analysis between δ^13^C time series of *N*. *pachyderma* and PC2 SLP 20CR over the North Atlantic domain: 2nd principal component from 20th Century sea-level pressure data [84], (i.e. the EAP), (B) Wavelet coherence analysis (WTC) between the two time series. The thick black contour designates the 5% significance level against red noise and the cone of influence where edge effects might distort the picture is shown as a lighter shade; the relative phase relationship is shown as arrows (with anti-phase pointing left), (C) Crossplot between the lowpass filtered *N*. *pachyderma* δ^13^C and PC2 SLP time series displaying the anticorrelation between the data; using the Stats Linear Correlation Test from IGOR PRO 6 software. The timeline displays that their antiphase relationship disappears from the 1950s onwards when the Marine Suess effect dominates. (D) Cross Wavelet Transform (XWT) analysis and (E) WTC between δ^13^C time series of *N*. *pachyderma* the surface easterly wind component (Vwind) of ERA-20C data of the 20th century from 1900–2010 [87] for winter months (NDJ) from a narrow 3 by 3-degree grid box (65–68 °N, 19–22 °W) North of Iceland over the core location; the relative phase relationship is shown as arrows (with in-phase pointing right), (F) Crossplot between the bandpass filters of *N*. *pachyderma* δ^13^C and the surface easterly wind component (Vwind) of ERA-20C data of the 20th century.

**Fig 7:**
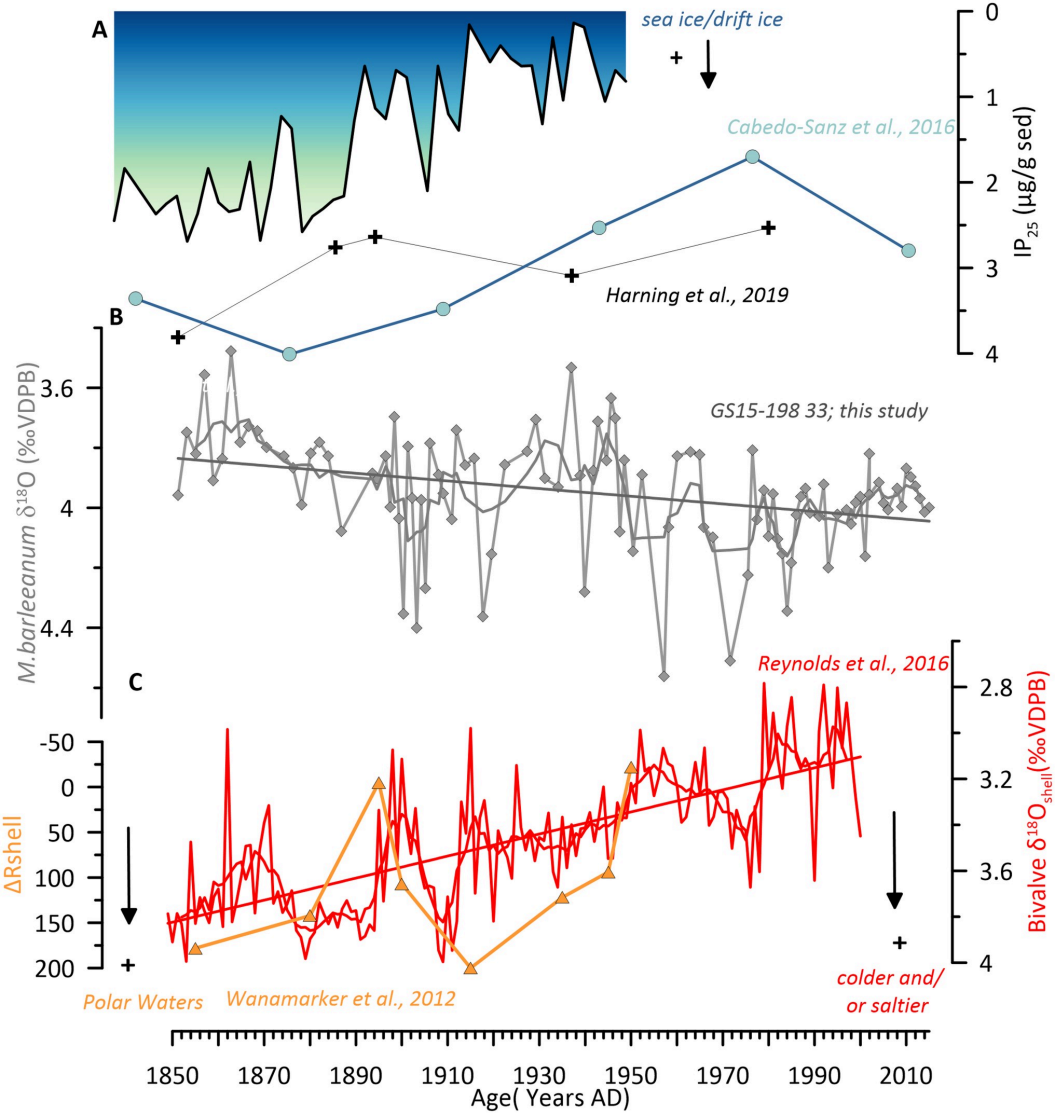
Comparison of selected marine climate proxies of core GS18-198-33 to other well-dated North Iceland shelf marine climate records. (A) IP_25_ values reflecting sea ice advection/ drift ice to the NIS [94–96]. (B) δ^18^O record of *M*. *barleeanum*. Trends are highlighted as linear regression lines. (C) ΔR in local schlerochronological records on the eastern NIS in orange and δ^18^O_shell_ record measured in *Arctic islandica* [25].

We initially present the core site and regional modern oceanography (Figs 1 & 2). Secondly, we will show the time series of stable isotopes for planktic and benthic species (Fig 3). That is followed by the comparison of the measured proxies against the instrumental data available in the area (Fig 4) and the evaluation of the Marine Suess effect at the core site (Fig 4). We discuss multi-decadal variability on the NIS and its dynamical forcing (Figs 5 and 6). Ultimately, we will place our results in a regional framework by comparing our data to other paleo-environmental records from the area to evaluate long-term trends (Fig 7).

### 1.1. Hydrographic setting: Modern oceanographic conditions

The NIS is an area where southern-derived Atlantic waters intercept with Arctic surface currents [28–30]. This sharp water mass boundary separates subsurface Subpolar Mode Water (SPMW) carried by the NIIC (5 to 8 °C) on the inner and mid-shelf [31] from the cool (1 to 4 °C) surface, low salinity and sea ice-bearing East Icelandic Current (EIC). The wedge of Atlantic Water on the NIS that sits between 100 and approximately 350–400m water depth, is cooled to 3.5°C and freshened to 34.9% [31]. The NIIC is a continuation of the Irminger Current (IC), which transports Atlantic and SPMW northwards along the western flank of the Reykjanes Ridge (Fig 1). Most of the IC turns south when Atlantic Waters reach the Denmark Strait, but approximately 5–10% flows northwards through the strait, and then turns eastwards and flows over the NIS [32] as the NIIC.

The NIIC overlies Upper Arctic Intermediate Waters (UAIW) (0–3 °C; S = 34.7–37.9, Fig 1B) [33], which occupies the water column down to approximately 500 m on the NIS [33–35]. AIW is mainly formed in the Greenland and Iceland Seas, and possibly in the Arctic Ocean [36]. It originates from Atlantic Water, which flows into the Nordic Seas, mainly over the Faroe–Iceland Ridge and through the Faroe–Shetland Channel [31] several years prior. In the Nordic Seas is exposed to atmospheric cooling and freshwater addition and ultimately overturns in the interior Greenland and Iceland Seas. This water mass then spreads out at intermediate depths of the Nordic Seas and makes up the bulk of the overflow water [35].

CTD (conductivity-temperature-depth profiler) casts were taken at each station during the ‘Ice2Ice’ project cruise GS 15–198 onboard the RV G.O. Sars research vessel in summer (July) 2015. The temperature, salinity, and oxygen profile at the specific core location (Fig 2), shows three layers, that characterize the water masses of different origins described above: relatively warm and saline Atlantic Water, cold low salinity Polar Water, and Arctic Intermediate Water [28, 37]. Based on the CTD profile we recognize a relatively fresh and warm surface layer with relatively high oxygen content. The EIC influences surface waters (< 25 m) at the core location [38]. The temperature drops steeply in a transition zone down to a depth of 75 meters. Between about 75–275 meters depth, there is a maximum in salinity at ~100 m. The oxygen content is lower (6.3–6.7 ml/l) than in the overlying layer. Temperature and salinity is characteristic of SPMW (>34.9 and >4°C) in that depth realm. The bottom part of the water profile, between approximately 275 and 350 meters is UAIW where temperature and salinity decrease, while the oxygen content fluctuates. The oxygen levels are higher (6.7–6.9 ml/l) than the above wedge of SPMW. Retrieved from 361 m water depth, marine sediment core GS15-198-33 sits in the UAIW (Figs 1B and 2).

## 2. Material and methods

The modern hydrographic data for the Iceland shelf used in this study to compare the proxy time series to was extracted for temperature from 1) the Marine and Freshwater Research Institute, Iceland for nearby stations to the core site (http://www.hafro.is/Sjora/), (S1 Fig in S1 File), and for phosphate concentrations (in a narrow grid cell from the database of International Council for the Exploration of the Sea (ICES) (https://ocean.ices.dk/https://ocean.ices.dk), (68–65 °N, 22–18 °W) (S2 Fig in S1 File). For the latter comparison, we selected CTD and Bottle data in a narrow grid cell (68°-65°N; 22–18°W) from the database and filtered according to calcification depth of the foraminifera and highest abundance season in the year (see 2.4. for more information).

CTD casts were also taken at each station during the cruise GS 15–198. Additionally, water sampling for δ^18^O of seawater, δ^18^O_sw_, and dissolved inorganic carbon δ^13^C of DIC, δ^13^C_DIC_ were conducted over the entire water column at all stations (Sect. 2.5.1); (S1 Table).

Sediment was collected using multi (MC) and gravity (GC) coring devices. In total, 6 MCs were taken at the coring station (MC-A to F). Approximately 43 cm of sediments were retrieved in the MCs and 4.07 m in the GC. In this study, MC-B and MC-E were used for analysis. Radionuclides (^210^Pb_unsupp_) were measured on both cores, MC-B and MC-E, and results indicate, a good agreement between these parameters justifying their combined use (S3 Fig in S1 File). The splicing between the MCs and the GC (gravity core) is based on a variety of parameters comprising foraminifera counting, mercury concentration, biomarker SST, carbonate concentrations, and radionuclide data obtained on both cores at a high resolution (see S4 Fig in S1 File). The splice for the composite depth /age was assigned to 12 cm in GC for MC-B and 15cm for MC-E (S2 Table). The data gives proof to an exact overlap between the MCs and the GC (S4 Fig in S1 File) in these centimeters.

### 2.1. Core location

The coring site, GS15-198-33, is located in the Húnaflóadjúp at a depth of 361 m (66°37,53 °N; 20°51,16 °W; Fig 1). Sub-bottom sediment property profiles indicated that this trough on the North Iceland margin facing the Iceland Sea contained a thick, drift-like sediment unit [39]. The unit is mainly deposited during the Holocene, and is about 40 km long and ca. 25–30 m thick.

### 2.2. Chronology

In this study, we use the age model published in [21], (S5 Fig in S1 File). Comprehensive dating of recent marine sediments offers the combined application of radionuclide dating (natural Lead - ^210^Pb, artificial: Caesium - ^137^Cs, Americium - ^241^Am) [40, 41], and of the environmental pollution marker mercury (Hg) [42, 43] (see S5 Fig in S1 File). This approach has been previously successfully used in the Black [44] and the Baltic Sea [45]. Support for the robustness of the age model presented in Perner, Moros [21] is provided by the similarity of variations in proxy data and historical/instrumental climatic records/data presented therein. Based on the evidence presented (for details please see supplementary information; (S3 Table)) we are confident that the age model of Perner, Moros [21] used here from a high-resolution open marine site is robust and of sufficient quality to allow conclusions regarding (paleo)oceanic changes on multi-decadal to partially decadal scale.

### 2.3 Sample preparation

Sediments from the MC-B and MC-E were sub-sampled at 0.5 cm resolution, whereas the sediments from the GC were sub-sampled at 1 cm resolution. Samples were wet sieved to >63 mm fraction with deionised water and then dry sieved to different size fractions for further analysis. Planktic foraminiferal assemblages counts [21] and their isotope analyses (this study) were conducted on MC-B whereas isotope measurements for benthic specie *Melonis barleeanum* were conducted on MC-E.

### 2.4. Foraminifera species ecology

1. The planktic foraminifer *Neogloboquadrina pachyderma* (*N*. *pachyderma*) (previously *N*. *pachyderma* (sinistral)); [46] typically calcifies, and records water properties, at ~100–200 m depth [47, 48]. A recent study based on Arctic and North Atlantic plankton tows shows that the depth habitat of *N*. *pachyderma* varies between 25 m and 280 m (average ~100 m) and this range of depth habitat is related to sea-ice and chlorophyll concentration [49]. In the Irminger Sea *N*. *pachyderma* reflects δ^18^O of the water without a significant offset from equilibrium at a calcification depth around 50 m and their abundances in the water column peak during the spring bloom (April-May) and in late summer (August-September) [50].
2. *Melonis barleeanum* (*M*. *barleeanum*) has an intermediate to deep infaunal microhabitat [51]. Stable isotope evidence suggests it prefers a rather static position within the sediment [52]. *M*. *barleeanum* is the most common and abundant species on the NIS [53] and is especially dominant on the outer NIS which has cool bottom waters of UAIW characteristics [54].

### 2.5. Stable isotopes

#### 2.5.1. Water and DIC isotope analyses

For δ^18^O_sw_ analysis, 60 ml serum vials with butyl rubber caps were filled with seawater during CTD operations on board. These were sealed and stored cool until analysis. For δ^13^C_DIC_ water analysis, 5 drops of 100% phosphoric acid (H_3_PO_4_) was added to the 12 ml exetainer vials using a 1 ml single-use syringe and flushed on the Gas Bench with Helium before ship departure at FARLAB laboratory. Then 1 ml of water sample was added into the exetainer vials during the cruise and stored at fridge temperatures (2–5 °C) before measuring samples after cruise return.

The stable isotope analyses for δ^18^O and δ^13^C_DIC_ in the seawater were performed on a Thermo Scientific Gasbench connected to a Thermo Scientific Delta V+ isotope ratio mass spectrometer at the Norwegian National Infrastructure FARLAB (Facility for advanced isotopic research and monitoring of weather, climate, and biogeochemical cycling) at the University of Bergen, Norway. The results are provided in the conventional δ-notation in ‰ relative to VSMOW for δ^18^O_seawater_ and VPDB for δ^13^C_DIC_. The analytical error of the measurements is 0.09 ‰ for δ^18^O, and 0.05 ‰ for δ^13^C_DIC_ (1 standard deviation).

#### 2.5.2. δ^18^O and δ^13^C of foraminifera calcite

Analysis for δ^18^O and δ^13^C in foraminifera calcite were also performed at FARLAB. The instrument used was an Finnigan MAT 253 mass spectrometer with an on-line coupled Kiel Carbon device. The results are defined in the conventional δ-notation in ‰ relative to VPDB for δ^18^O calcite. The analytical precision of the measurements is better than 0.06‰. Replicate measurements for *N*. *pachyderma*, conducted when enough material was available, yielded an average 1 standard deviation of 0.14 ‰ for δ^18^O and 0.15 ‰ for δ^13^C (n = 45). The size fraction 106–350 μm was used for planktic and benthic stable isotope analysis.

Estimates of equilibrium δ^18^O (δ^18^O_eq_) in the water column for the foraminiferal species used in this study, were derived from the equation of Kim and O’Neil [55]. We substituted water temperature and δ^18^O_sw_ with actual CTD and water isotope measurements from the GS15-198 cruise:

T=16.1-4.64((δ18Oc-δ18Osw)+0.09(δ18Oc-δ18Osw)^)2 (1)

where δ^18^O_c_ and δ^18^O_sw_ are the δ^18^O of calcite and seawater, accordingly. We use the -0.27% conversion of Hut [56] to convert δ^18^O_seawater_ Standard Mean Ocean Water (SMOW) to δ^18^O_seawater_ VPDB.

δ18Osw=δ18Oc-0.27‰ (2)

### 2.6. Spectral analysis

Classical frequency analysis was employed on the isotope data from core GS15-198-33, on each record using the "redfit" function [57] embedded in the “dplR” library in R [58]. The routine computes red-noise corrected spectrum time series using the Lomb-Scargle Fourier transform and tests significance levels against the theoretical spectrum based on simulated AR1 series (i.e. with spectral characteristics similar to those of the isotope record). All the time series have been bandpass filtered to remove the high-frequency variability (higher than 5 years) and linearly detrended–a common procedure for oceanographic time series.

For correlations between the proxy data and the instrumental time series used in this study, age uncertainties were taken into account. To estimate the p value, we carried out an autocorrelation estimate and determined the lag correlation with the e-folding time of the autocorrelation function. Significance levels were inferred via correlation of the proxy time series to 10k red noise realizations with spectral characteristics similar to the instrumental data following the rationale from [59]. For each Monte Carlo iteration, we propagated the chronological error using a randomly resampled and monotonically increasing age model within the error associated with the CFCS ^210^Pb measurements (S5 Fig in S1 File).

Additionally, to investigate the temporal evolution of periodic and transient signals identified using the frequency analysis above, we performed continuous wavelet transform (CTW) analysis on our isotope records following the method described in Grinsted, Moore [60]. The wavelets were calculated using the l analytic Morlet wavelet and significance levels were tested against the theoretical red-noise (AR1) background spectrum. The statistical significance level of the wavelet coherence is estimated using Monte Carlo methods. Prior to wavelet analysis, time series were linearly interpolated at a 1-year resolution. To identify common signals and their temporal evolution between time series we performed cross wavelet transform (XWT) and wavelet coherence (WTC). The XWT exposes regions with high common power and further reveals information about the phase relationship [60]. The WTC between two CWT can find significant coherence even though the common power is low [60].

The filtering on the *N*. *pachyderma* δ^13^C time series shown in Fig 6D and 6G was performed using a Butterworth filter (Matlab inbuilt algorithm) with a 15 order lowpass filter and a normalised cutoff frequency at 0.1 and a 10 order lowpass filter with a normalised cutoff frequency at 0.04. See S6 Fig in S1 File for the bandpass filter response function. The filtering of the time series on specific bandwidths is based on the significant frequencies identifies in the classical frequency analysis (Fig 3).

## 3. Results

The δ^18^O composition of planktic species *N*. *pachyderma* (1850–2015 AD) ranges from values between 2.0 to 3.7‰ (VPDB) with a mean value of 2.9 ‰ (Fig 3A). Our CTD profile, a snapshot in July 2015 conditions, indicates that the calculated δ^18^O_eq_ (VPDB) of *N*. *pachyderma* occurs beneath the fresh surface layer, at a salinity maximum around 75–125 m (Fig 2).

Classical frequency analysis indicates a 16-year periodicity in the δ^18^O *N*. *pachyderma* however, it is not significantly above the red-noise spectrum (87% CL) (Fig 3E). Notwithstanding, wavelet power spectrum analysis indicates that the 16-year periodicity is significant with a center around the mid 20^th^ century (S7 Fig in S1 File). The reason why this frequency is not evident in the classical spectral analysis might be related to the fact that this type of analysis is an average so it might smooth out localised cyclicities that one would see on the other hand in the wavelet spectrum.

Variability in the δ^13^C shell record of *N*. *pachyderma* spans from 0.4‰ to -0.3‰ (VPDB) and is dominated by multi-decadal variability with a decreasing trend especially since the 1940s/1950s (Fig 3B). The overall shift to lower values is -0.7‰ (VPDB) over the 1900–2000 AD time period (Fig 3B). Classical spectral analysis displays a significant (95% CL) ~ 4- yr and ~ 30-yr frequency in the δ^13^C time series (Fig 3E). The amplitude of the shift of this ~ 30-yr cycle is ~ 0.6‰ (Fig 3B). Wavelet analysis confirms the significant ~ 30-yr period, but displays that this frequency is only dominant until the 1940s/1950s (S8 Fig in S1 File).

The δ^18^O record of benthic foraminifera *M*. *barleeanum* ranges from 3.2–4.2‰ (VPDB) over the 1850–2015 AD time period with the lowest values around 1860 AD (Fig 3C) and a visible long-term trend to more δ^18^O enriched values towards the present day. We notice an average (n = 2) 0.33 ‰ offset between the measured core top values for benthic species *M*. *barleeanum* and the calculated δ^18^O_eq_ at bottom water depth at the core site (361m = 4.02‰, (VPDB)); (GS198-33MC-B 0–0.5 cm = 3.71‰, (VPDB); GS198-33MC-E 0–0.5 cm = 3.67‰, (VPDB)). Kristjánsdóttir, Lea [61] found very similar offset values (+0.27‰ offset) for *M*. *barleeanum* in a nearby study. This is likely due to vital effects/disequilibrium. Benthic foraminifera construct (precipitate from seawater) their CaCO_3_ shells either in isotopic equilibrium or inconsistent isotopic disequilibria with the seawater in which they live [62]. Such disequilibria "vital effect" may be due to the influence of respiratory CO_2_, but appear unrelated to environmental conditions such as depth or temperature [63]. We use disequilibrium corrected δ^18^O values for *M*. *barleeanum* in this study.

Classical spectral analysis displays a significant (95% CL) ~ 7-yr frequency in the benthic δ^18^O record (Fig 3F). Wavelet power spectrum analysis using the Morlet wavelet method reveals a significant 16-year periodicity in the δ^18^O of *M*. *barleeanum* that is mostly present 1890–1980 AD (S9 Fig in S1 File).

Stable carbon isotope values range from -1.6‰ to -0.4‰ (VPDB) for *M*. *barleeanum* (Fig 3D) Classical spectral analysis displays a significant (95% CL) ~ 4-yr peak in the δ^13^C time series (Fig 3F)- a frequency that is shared with the δ^13^C record of *N*. *pachyderma*.

## 4. Discussion

### 4.1 Comparison of foraminifera proxy data and instrumental records

It has been argued that around Iceland variations in δ^18^O_c_ of planktic foraminiferal species over the last 10 ka are principally controlled by temperature variations while salinity changes prove less important [64]. Conversely, the δ^18^O-shell record of the marine bivalve mollusc *Arctica islandica* collected equally from the NIS was considered to represent seawater density changes, which is the combined product of seawater temperature and salinity [13, 25].

To test these relationships for our time series the *N*. *pachyderma* δ^18^O data is compared to nearby instrumental data (Hunafloi section; station 3, HF 3: 66.5 N; 20.78 W; S1 Fig in S1 File); (Fig 4A) obtained during the season of highest abundance/flux of this species (May-August). Continuous instrumental data for the Hunafloi section is only available after 1974, hence we use data from the Siglunes section, situated further east and operated since 1947 (SI3: 66.32 N; 18.50 W; S1 Fig in S1 File) for the comparison in the older part of the record.

The instrumental temperature data at around 100m-water depth which correspond to the estimated calcification depth of *N*. *pachyderma* in this study, and the *N*. *pachyderma* δ^18^O series are anticorrelated (*r* = -0.38; sd = 0.06; and *r* = -0.44: sd = 0.07 on 5-year high-pass filtered data: *p*<0.01). High δ^18^O values occur in cold years in the instrumental record and vice versa (Fig 4A). The oxygen isotope record shows high δ^18^O values, up to 3.7 ‰ (VPDB), in the late 1960s, (Fig 4A), which indicates conditions that are colder than the years before and after. This is matched by the instrumental data at the nearby Hunafloi station where temperatures drop by 6 °C during that interval (Fig 4A). It is well known that during the “Great Salinity Anomaly” (GSA), Polar waters dominated the NIS over the entire water column [34, 66]. At this time the hydrographic conditions in North Icelandic waters changed from being Atlantic (t>4°C; S>35.0) to Polar conditions (t~0°C; S as low as 34.0) [67].

Southward shifts of the Polar Front caused by outbursts of cold and fresh waters from the Arctic Ocean, were commonly seen during the GSAs from the 1960-1990s [68]. The timing in our data fits well with the start of the GSA in the late 1960s and suggests that it reached the NIS via increased inflow of the EGC/EIC and not via the NIIC [66]. Oceanographic conditions in Icelandic waters are closely related to atmospheric forcing, in particular prevalence of northerly vs. southerly wind directions in the Denmark Strait [69]. Strong northerly winds prevailed during the GSA due to a positive Mean Sea Level Pressure (MSLP) gradient across the Denmark Strait, with a high pressure over Greenland [69]. Northerly winds result in a reduced northward volume flux of Atlantic Water in the Irminger Sea and ultimately onto the NIS. Moreover, the prevailing northerly winds resulted in a stronger southward flow of Polar Waters within the EGC causing cold conditions North of Iceland and drift ice blocking the north and east coasts of the country [66]. In the following years in 1975–1979, 1982 and 1988 Polar Water also dominated the Shelf [34]. Shifts to high δ^18^O values indicating colder conditions can again be identified during these years however they are less pronounced compared to the one in the late 1960s. Moreover, single years e.g. 1982 and 1988 are difficult to identify precisely given that we do not have yearly resolution throughout the record.

We acknowledge that foraminiferal δ^18^O ideally best represents hydrographic variability and thus should reflect the combined variations in seawater temperature and salinity. If our *N*. *pachyderma* δ^18^O series was mainly salinity-driven, we would expect that our data show the opposite sign of direction (lower δ^18^O) during low salinity events (such as the GSA). However, it displays higher δ^18^O values ie. colder conditions rather than fresher ones (Fig 4A). Moreover, the comparison between instrumental salinity and derived δ^18^O_sw_ of *N*. *pachyderma* (not shown) held no significant correspondence. Hence, we conclude, that our time series of *N*. *pachyderma* δ^18^O seems to be more controlled by temperature variations than salinity over the studied time period in line with previous studies on foraminifera [64].

Phosphate and δ^13^C have an inverse relationship, hence low-nutrient, low phosphate water masses have a high δ^13^C signature, and vice versa [70]. To evaluate this relationship in North Iceland waters the *N*. *pachyderma* δ^13^C data is compared to historical oceanic PO_4_ data (Fig 4B). Trends in our *N*. *pachyderma* δ^13^C_foram_ record match the measured phosphate concentration (Fig 4B) in the upper 200 m well on the NIS (66–68 °N, 18–22 °W; S2 Fig in S1 File) between April and September (representing the highest abundance season and calcification depth range of *N*. *pachyderma*) from 1950s to 2000s (Fig 4B). Overall, low phosphate low nutrient values match high δ^13^C values and inversely. During the GSA in the late 1960s more polar EGC-sourced waters reached the NIS. In general, waters from the polar domain are cold and nutrient-depleted, hence would have a high δ^13^C signature [71]. A North-South transect of phosphate concentrations (μmol kg ^−1^) along the EGC over the Greenland-Scotland Ridge in the Denmark Strait based on the GLODAPv2 data [72] shows that waters at approximately 100m water depth north of the sill have lower phosphate levels compared to south of the ridge (S10 Fig in S1 File). This southern region is influenced by the contribution of the Irminger Current contributing surface waters with higher levels of nutrient (hence higher phosphate concentrations) to the water column.

During the GSA a shift to lower nutrient levels of the Atlantic-layer should be accompanied by a drop in temperature and salinity. Indeed, our proxy data displays higher *N*. *pachderma* δ^13^C values, indicative of lower nutrients and higher δ^18^O values, characteristic for colder waters confirming that the foraminiferal signal reliably reflects a water mass shift at that time in line with the historical observations (Fig 4A & 4B). Moreover, between 1975–1979 Polar Waters dominated the NIS recurrently [34] and we also document higher δ^13^C values but less pronounced than in the late 60s potentially related to the lower sampling resolution (Fig 4B).

Other time intervals, however not covered by the instrumental observations that have evidenced greater dominance of colder Polar Waters on the NIS are centered around 1880s and 1910s [25, 73], (for reference also see Fig 7C). These periods correspond to high δ^13^C values in the *N*. *pachyderma* record implying low nutrient waters (Fig 4B). Based on that evaluation, increasing δ^13^C shifts in *N*. *pachyderma* overall record changes in the relative proportion of Polar Waters entrained into the NIS via the EIC as for example during the GSA.

### 4.2. Foraminifera trace anthropogenic CO_2_ in the Iceland Sea by ~ 1950

The decreasing trend identified in the *N*. *pachyderma* δ^13^C record since the 1950s is consistent with the Marine Suess effect (Fig 4B and 4C) [26]. The most pronounced shift to lower values in our data is observed from the 1950s onwards. This might be attributed to the more intense burning of fossil fuels and steeper decrease in atmospheric δ^13^C and hence incorporation into the ocean since then [65] (Fig 4C). However, we notice that the absolute amplitude shift in our data is smaller than what can be estimated at 100 m water depth at the core location (grid point 66.5N, 20.5W) (Fig 4C) based on the mapped Nordic Seas fields [74] and the atmospheric δ^13^C record from Rubino, Etheridge [65]. These studies show that the estimated Suess effect at 100 m in 2010 should be around ~-1‰ (VPDB) (Fig 4C). The calculation of the Marine Suess effect in the NIS waters is based on a transient steady-state assumption i.e. no changes in circulation and biology. This process involves the following two steps (1) finding the ratio between atmospheric and ocean Suess effect in 2012 (the nominal year of the maps shown in Eide and Olsen [74]) in the grid cell and depth level of interest, and then (2) multiply the time series of atmospheric Suess effect with this ratio to derive the ocean Suess effect records.

Our observation suggests that the foraminiferal δ^13^C records an attenuated Suess effect compared with the calculated seawater estimates at the core location (Fig 4B and 4C). The results replicate the subdued Marine Suess effect that has previously been observed in proxy studies on the NIS based on the marine mollusk *Arctica Islandica* [75, 76]. Atmospheric stable carbon isotope values and that of marine mollusks can often be decoupled from each other [76]. Moreover, it has been noticed that there is an offset among δ^13^C curves of marine mollusks from different locations indicating different degrees as to how the Suess effect is incorporated into shells during their life cycle [76].

Here, we attribute the reduced amplitude shift in the Suess effect to the fact that this process is counteracted by a contemporary ongoing increase in surface ocean productivity since the 1940/50´s (Fig 4D). That would cause a shift in the foraminiferal δ^13^C towards higher values (Fig 4B). Thus counteracting the full magnitude of an expected ~-1‰ decreasing trend (Fig 4B). This agrees with a recent study on the same core material that finds that Greenland’s freshwater discharge has contributed to a nutrient-driven fertilization of the upper ocean and consequently increased the marine primary productivity since the 1940s/50s on the NIS [21]. The increased productivity signal is well displayed in the carbonate content of the sediment and the planktic foraminifera flux rates and is well in line with the onset of the attenuated Suess effect in our δ^13^C record (Fig 4B–4D). Based on these considerations, we chose to not correct the planktic δ^13^C record for the Marine Suess effect as done elsewhere [e.g. 75].

Another possible mechanism that could explain the attenuated marine Suess effect captured by our δ^13^C record is related to mixing processes that bring relatively older, less equilibrated AIW source waters into the surface layer. The strength of the marine Suess effect signal is strongly linked to the degree in which the source waters are equilibrated with the atmosphere [77]. AIW source waters are less equilibrated with the atmosphere than SPMW [24]. The reduced level of equilibrium with the atmosphere would therefore lead to a reduced marine Suess effect signal in the foraminiferal shells. For that scenario to be valid, a long-term trend since the 1950s for a shift to AIW dominating on the NIS within the water column at ~100–200 m depth (where *N*.*pachyderma* typically calcifies and records water mass properties) should be identified, but this is, however, not supported by observations [17, 25]. Neither does the local wind forcing data since the 1950s over the core site support evidence for a contemporaneous long-term shift that would permit stronger in-situ upwelling of AIW to the surface (see chapter 4.3.2. for reference on wind-forcing).

Taken together, more studies within the Nordic Seas are required to better constrain the precise timing and understand the underlying reason for the rate of ocean δ^13^C DIC decline in response to the observed shift in atmospheric δ^13^C. This information is lacking to quantitatively correct time series for the Marine Suess effect appropriately.

### 4.3. Reconstructed Inter- and multi-decadal climate variability on the NIS

#### 4.3.1 Poleward propagation of SST anomalies in the Nordic realm

Wavelet power spectrum analysis of our data highlights a 16-year periodicity in the δ^18^O *N*. *pachyderma* profiles that indicate high-frequency variability in temperature in the Atlantic Water layer (Fig 3A, S7 Fig in S1 File). This interdecadal variability is consistent with previously observed atmosphere-ocean variability in the subpolar North Atlantic [78, 79] and the Nordic Seas [80]. This frequency of climate variations is likely linked to the poleward propagation of temperature (salinity) anomalies in the pathway of the North Atlantic Current (NAC) and its poleward extension, the Norwegian Atlantic Current (NwAC). A recent study showed that this dominant 14–16 year frequency identified in the ocean is also apparent in variations of atmospheric circulation [81]. Similarly, considerable decadal-scale variability is observed for surface air temperature (SAT) and precipitation over Norway, and in winter Arctic sea ice extent. Higher Norwegian Sea SST is associated with higher SAT and increased precipitation whereas reductions in sea ice extent lag increasing SST by 3 years.

We compared our time series of δ^18^O *N*. *pachyderma* to the data sets of Hadley Centre SST (HadISST) [82], which is provided on a 1°grid, and with a temporal resolution of 1 month as presented in Årthun, Eldevik [81], (Fig 5A). Comparing the SST data along nine defined stations (St) from South to North within the pathway of NAC and NwAC with our δ^18^O data applying cross wavelet transform and wavelet coherence analysis [60] we find the best correspondence with the 4 southernmost stations (Fig 5B–5G). The constructed Cross Wavelet Transform (XWT) analysis between the two time series exposes their significant common power in the 16-year band from 1940–1960s, (Fig 5F) for St1-4. Moreover, the wavelet coherence (WTC) finds significant coherence in the common 16-year band. Compared with the XWT, a larger section stands out as being significant especially in comparison with St2 and 3. The coherency between our dataset and the southernmost station from Årthun, Eldevik [81] can be explained by a common influence from the South as an expression of variability in the northward-flowing Atlantic Waters via two of the inflow branches across the Denmark Strait and the Greenland-Scotland Ridge into the Nordic Seas and ultimately Arctic Ocean [3].

This is the first study, to date, that has identified that this inter-decadal frequency of poleward propagation of temperature anomalies along the NAC and NwAC also influences the area Northwest of Iceland via the Iceland inflow branch. The high common power between the time series centered in the 1940–1960s might be due to a general larger Atlantic Water influence during this period. The years around 1950 seemed to be favoured by the century’s greatest supply of Atlantic Water to the NIS due to a negative Mean Sea Level Pressure (MSLP), i.e. more southerly winds across the Denmark Strait [34].

We also note a common power with the northernmost station 9 off Svalbard in the northern Nordic Seas in the XWT analysis (Fig 5G) that is, however, not revealed in the WTC (Fig 5F). Cross-correlation between δ^18^O of *N*. *pachyderma* with St9 SST´s between 1950–2015 AD is significant (*r* = -0.25; sd = 0.11; *p*< 0.05; on the 16-yr high-pass filter *r* = -0.54; sd = 0.15; *p*< 0.05). The coherency between our dataset and the northernmost st9 could potentially arise from the influence of recirculating Atlantic Waters that are entrained into the East Greenland Current from West of Spitsbergen and the Atlantic layer in the Arctic Ocean [83]. These water masses would reach our core site via the EGC/EIC and explain the common temporal variability observed in these areas.

Interestingly, we also find that the 16-year periodicity (with center from 1890–1980 AD) is equally observed in the AIW based on the *M*. *barleeanum* δ^18^O profiles (S9 Fig in S1 File). That implies that years of stronger SST anomalies into the Nordic realm within the pathway of NAC and NwAC equally impact the intermediate waters North of Iceland. Years of enhanced Atlantic Water inflow into the NIS result in the expansion of the warmer and more saline core of the NIIC within the water column, ultimately penetrating the deeper intermediate-depth intervals. Thus, shifting the water mass boundary layer/front between SPMW and AIW to deeper levels.

Finally, the spectral analysis displays a significant (95% CL) ~ 7-yr frequency in the benthic δ^18^O record (Fig 3F). A sub-decadal ~8-year frequency, which is also evident in the NAO records [79], has also been identified in the Norwegian Sea SST and is associated with higher SAT [81]. This might hint at the fact that the poleward propagation of ocean temperature anomalies along the NAC-NwAC pathway is a common feature influencing multiple branches of Atlantic inflow into the Nordic Seas also on sub-decadal timescales.

#### 4.3.2 Local wind-driven upwelling processes on the Northwest Icelandic shelf

Wavelet analysis revealed a significant ~30-yr period in the δ^13^C of *N*. *pachyderma* (S8 Fig in S1 File). Here, we interpret this ~ 30-yr variability as a regular cycle between more enriched and depleted δ^13^C associated with local wind-driven upwelling of AIW to the near-surface. To test the hypothesis that a proportion of the variability captured by the δ^13^C data is associated with atmospheric forcing and the resulting prevailing winds over the NIS, we performed correlation analysis with the PC2 SLP 20CR over the North Atlantic domain: 2nd principal component from 20th Century sea-level pressure data [84], i.e. the East Atlantic (EA) Pattern [85] (Fig 6).

The EA pattern is the second dominant mode of atmospheric circulation in the North Atlantic [86] and has its centers of action-oriented in a West-East direction [85], i.e. a negative EA pattern is usually associated with higher pressure/more frequent blocking circulation over Northern Europe/Scandinavia. The XWT analysis between the two time series exposes their significant common power in the ~ 30-yr band from 1900–1940s, (Fig 6A). Moreover, the WTC finds significant coherence in the common 30-year band and a larger time period stands out as being significant (Fig 6B). We find a significant anti-correlation between our *N*. *pachyderma* δ^13^C record and the EA pattern (*r* = -0.56; SE = 0.24; *p*< 0.05), suggesting that lower δ^13^C corresponds to higher PC2 SLP values i.e. indicating more intense northeasterly winds over the Nordic Seas (and vice versa) (Fig 6C; S11 Fig in S1 File).

To define specifically the role of the local wind forcing, we compared the surface easterly wind component of historical reanalyses data set ERA-20C: ECMWF’s atmospheric reanalysis of the 20th-century data set [87] North of Iceland with our δ^13^C time series (Fig 6D–6F). The XWT analysis between the two time series exposes their significant common power in the ~ 30-yr band from 1900–1940s, (Fig 6D). Moreover, the WTC finds significant in-phase coherence in the common 30-year band throughout the entire period standing out as being significant (Fig 6E).

Correlation between the bandpass filtered easterly wind component from ERA-20C and the bandpass filtered δ^13^C data in the interval from 1903–1963 was strongly significant (*r* = 0.79; SE = 0.16; *p*< 0.001), (Fig 6F). The comparison between surface easterly wind component of the twentieth Century Reanalysis (20CR) data and the bandpass filtered δ^13^C data is shown in S11 Fig in S1 File for reference.

In general, periods of reduced zonal winds stress along the North Icelandic Shelf, indicating an easterly flow anomaly (as the normal zonal wind direction is westerly) co-vary with lower δ^13^C values (Fig 6F). On the NIS, along-shore winds and Ekman circulation would cause upwelling and downwelling. Easterly winds generate offshore Ekman transport. This offshore transport of mass in the surface layer is balanced by an onshore flow at depth and an Ekman pumping of water from depth into the surface layer, which would cause the upwelling of AIW onto the shelf. The wind-induced upwelling in connection with a stronger easterly flow of ^12^C-enriched AIW into the shallower surface waters could shift the carbon isotope signature to lower values as observed in the *N*. *pachyderma* data. Indeed, the measured water δ^13^C_DIC_ at the core location in July 2015 has lower δ^13^C values in agreement with the presence of deeper AIW (Fig 2). Moreover, contributions from bottom waters deeper than 500 m water depth (Fig 1C) could be an additional source of more depleted waters explaining the shifts to lower values (Fig 1C). Conversely, higher δ^13^C values are probably related to shifts in the relative proportion of more Polar Waters entrained onto the NIS as for example during the GSA as discussed earlier in section 4.2.

One example is the GSA when the δ^13^C and δ^18^O vary together (Fig 4A and 4B). However, we do not find a persistent 30-yr periodicity in the δ^18^O as seen in the δ^13^C record. This δ^13^C and δ^18^O discrepancy remains challenging to explain as one would expect that changes in upwelling rates would affect both the temperature and salinity continuously. One potential explanation for the δ^13^C and δ^18^O inconsistency might be related to the timing when both signals are incorporated into the foraminifera shell throughout their life cycle. The mixing by winter storms brings nutrients to the surface. Most of the biological production occurs during ephemeral spring blooms in the Nordic Seas lasting only a few weeks [88]. Hence the incorporation of the “nutrient signal” as δ^13^C in the shell might be rather rapid and may not necessarily correspond to the timing (seasonal) when the temperature and salinity signal is formed. Moreover, the gradient in δ^13^C of the water column is rather steep in the upper layer/thermocline compared to the observed temperature profile at the same depth (Fig 2). This means that a shift of upper, EIC derived Polar Waters to the depth realms where *N*. *pachyderma* calcifies would cause a more pronounced response in the δ^13^C signal however not necessarily in the δ^18^O at the same time due to their differing slopes.

During photosynthesis, organisms preferentially take up the lighter isotope of carbon (^12^C) increasing surface ocean δ^13^C. It could thus be assumed that periods of stronger inflow of SPMW to the core site triggers increased primary production due to increased nutrient levels, which would shift the planktic δ^13^C signal to higher values. As previously discussed, the inflow of southern sourced waters onto the NIS is believed to be wind-driven with lower MSLP allowing more southerly flow and Atlantic Water influence [69]. Conversely high δ^13^C values in our data are recorded during times when low-nutrient Polar Waters dominated the site (e.g GSA) hence precluding that Atlantic–water derived productivity changes trigger these shifts to higher values.

As for temperature, δ^13^C values also depend on the carbonate chemistry of seawater with a decrease in foraminifera δ^13^C under increasing CO_3_^-2^ concentration of the surrounding seawater [89, 90]. Temperature and CO_3_^2^ properties covary in the water column; hence it is difficult to distinguish their respective influences on foraminiferal δ^13^C. Conditions of higher pH (and CO_3_^-2^) cause significant changes to the ambient chemistry of the foraminiferal microenvironment and most likely lead to changes in the shell δ^13^C composition. Increases in CO_3_^-2^ cause the δ^13^C in foraminifera to become depleted relative to δ^13^C DIC [89]. Interpreting the high δ^13^C signature in our data set as more Polar Water influence on the core site would require accounting for the CO_3_^-2^ influence on the foraminiferal δ^13^C. Polar-derived water masses have lower pH hence lower CO_3_^-2^ concentration (S12 Fig in S1 File), which would cause the δ^13^C in foraminifera to become more enriched in ^13^C. The same direction shift to higher values would be caused by the low temperature of Polar Waters due to the temperature sensitivity of ~0.17%/°C of foraminiferal δ^13^C [91]. However, similar to the temperature effect even if the δ^13^C variability was purely a result of CO_3_^-2^ driven fractionation processes it would still indicate a change in the water mass at the core site.

In conclusion, we propose that the observed ~30-yr variability in the planktic foraminiferal δ^13^C reflects alternating dominance of local wind-driven upwelling processes of AIW towards the subsurface (low δ^13^C values) and cold/fresh and nutrient-poor, Polar Waters (high δ^13^C values).

### 4.4. Climate change during the historical time period: Variability of subsurface and intermediate waters on the NIS

The NIS has been targeted for investigating paleoceanographic variability extensively over the past decades. Most studies have targeted the long-term trends over the Holocene [e.g. 92] and/or over the past 2,000 years [93 and references therein].

Here, we target a regional comparison to evaluate long-term patterns over the instrumental time period: 1850–2015 occurring superimposed on the identified multi-decadal variability. One way to achieve that is by comparing our time series to the existing proxies from the NIS such as local schlerochronological [24, 25] and drift ice records [94–96] which are sensitive to changes in surface and subsurface water conditions in this area linked to the varying influence of Polar- and Atlantic sourced currents. For the comparison, we chose the δ^18^O record of *M*. *barleeanum* from this study as it displays an obvious long-term trend towards higher δ^18^O values from 1850- to 2015. We interpret that shift as increased salinities in the intermediate waters at the core location over time. Below, we will explore a possible interpretation for that long-term trend that is in line with and supported by the trends displayed in the other proxy records from the Shelf over the same time period.

Sea ice proxies such as IP_25_ [97–99] show a long-term shift towards lower IP_25_ concentrations from 1850 to 2015 AD (Fig 7A) which implies reduced sea ice advection/ drift ice to the NIS [94–96]. Increased IP_25_ has been interpreted to suggest greater dominance of colder Arctic surface waters [95 and ref. therein]. Hence, the observed decline indicates reduced sea ice advection/ drift ice to the NIS and a long-term shift towards an Atlantic Water dominated regime over the 20^th^ century on the NIS.

This is in line with our benthic δ^18^O record that displays a long-term trend to higher values towards present-day suggesting a core shift of more saline waters of the NIIC into deeper intermediate depths (Fig 7B). That pattern is mirrored by the long-term trends over the same time period captured in the lower δ^18^O_shell_ and a reduction of ΔR in local schlerochronological records on the eastern NIS (Fig 7C), [23, 25, 73, 100]. This indicates the increased presence of warmer Atlantic waters on the NIS in the subsurface over the historical era.

A trend towards more saline Atlantic Water and heat being transported onto the NIS is also documented in the instrumental record between 1994 and 2010 [17, 101]. The latter results in a (seasonal) core shift of the warmer more saline Atlantic Waters of the NIIC to the deeper intermediate shelf areas North of Iceland that were previously occupied by AIWs. The stronger and warmer Atlantic inflow will cause the core of the NIIC to expand which will ultimately shift the SPMW/AIW boundary to deeper realms within the water column as documented in our data set.

## 5. Conclusions

Extending oceanographic data beyond the instrumental period is required to better characterize and understand multi-decadal to centennial natural ocean variability. This study demonstrates that this can be established using well-dated marine sediments from the NIS.

The isotopic signatures of *N*. *pachyderma* on the NIS reflects temperature at around ~100 m in the water column, hence recording Atlantic–sourced water mass variability. Shifts in the δ^13^C signature of *N*. *pachyderma* matches the measured phosphate concentration in the upper 200 m on the NIS overall well.

Inter- and multi-decadal climate variability is identified. We demonstrate that a 16-year periodicity is present in SPMW and AIW based on the δ^18^O data of *N*. *pachyderma and M*. *barleeanum*. That implies that years of stronger SST anomaly propagation into the Nordic Seas within the pathway of the NwAC equally impacts other Atlantic Water inflow branches such as the Icelandic one via the Denmark Strait.

The reconstructed North Icelandic *N*. *pachyderma* δ^13^C has shown strong variability with multi-decadal cycles (30 years) comparable to those identified in the records of the EAP. We interpret the ~30-yr variability as a regular periodicity reflecting alternating dominance of local easterly wind-driven upwelling processes of AIW (low δ^13^C values) and advection of more cold/fresh and nutrient-poor, i.e. when Polar Waters dominate the Shelf (high δ^13^C values). Enhanced export of freshwater and sea ice from the Arctic is evident in our *N*. *pachyderma* δ^13^C values during times when low-nutrient Polar Waters dominated the site as during the GSA, as well as around 1880s and 1910s [24, 25].

Despite the hydrographic variability identified, the carbon isotope (δ^13^C) time series of fossil foraminifera reveals a decreasing δ^13^C excursion driven by anthropogenic CO_2_ penetration into the surface ocean, the “Suess effect” signal. We find evidence that the fossil fuel-derived CO_2_ uptake in the NW Icelandic Shelf area emerges in ~1950 ±8. However, it can be noticed that the absolute amplitude shift in the data is smaller than the estimated ~-1‰ shift at the water depth at the core location. This reduced amplitude shift in the Suess effect indicates that this process is counteracted by a contemporary ongoing increase in surface ocean productivity since the 1940/50´s causing a shift in the foraminiferal δ^13^C towards higher values. Thus counteracting the full magnitude of an expected ~-1‰ decreasing trend. This mechanism would agree with a recent study on the same core material [21] that finds that enhanced Greenland’s freshwater discharge has contributed to a nutrient-driven fertilization of the upper ocean and consequently increased the marine primary productivity since the 1940s/50s on the North Icelandic Shelf.

The regional comparison of the long-term trend in the *M*. *barleeanum* δ^18^O data in this study with other records from the area all together show reduced sea ice advection/drift ice to the NIS from 1850 to 2015 AD. That follows the increased presence of warmer Atlantic waters on the shelf between 1850–2015 AD. This in turn causes a core shift of warmer/saline waters transported by the NIIC to penetrate deeper into intermediate depths than observed before.

## Supporting information

S1 Table Water isotope data for oxygen and DIC from July 2015 at core location. (XLSX)

S2 Table Planktic+benthic foraminifera stable isotope data on original depth/corrected depth/splice depth/age. (XLSX)

S3 Table Age-depth model with uncertainties. (XLSX)

S1 File (DOCX)
