# Supplementary material for: A multi-decadal record of oceanographic changes of the past ~165 years (1850-2015 AD) from Northwest of Iceland
Source: PLoS One. 2020 Sep 29;15(9):e0239373. doi: 10.1371/journal.pone.0239373 (PMC7523958; doi:10.1371/journal.pone.0239373)
Supplement: S1 File — (DOCX) [file pone.0239373.s004.docx]

A multi-decadal record of oceanographic changes of the past

~165 years (1850-2015 AD) from Northwest of Iceland.

Margit H. Simon (1)* Francesco Muschitiello (2,1) Amandine A. Tisserand (1) Are Olsen (3) Matthias Moros (4), Kerstin Perner (4), Siv Tone Bårdsnes (5), Trond M. Dokken (1), Eystein Jansen (5,1)

(1) NORCE Norwegian Research Centre, Bjerknes Centre for Climate Research, Norway; (2) Department of Geography, Cambridge University, UK; 3) Geophysical Institute, University of Bergen, Bjerknes Centre for Climate Research, Norway; (4) Leibniz Institute for Baltic Sea Research, Department of Marine Geology, Germany; (5) Department of Earth Sciences University of Bergen, Bjerknes Centre for Climate Research, Norway.


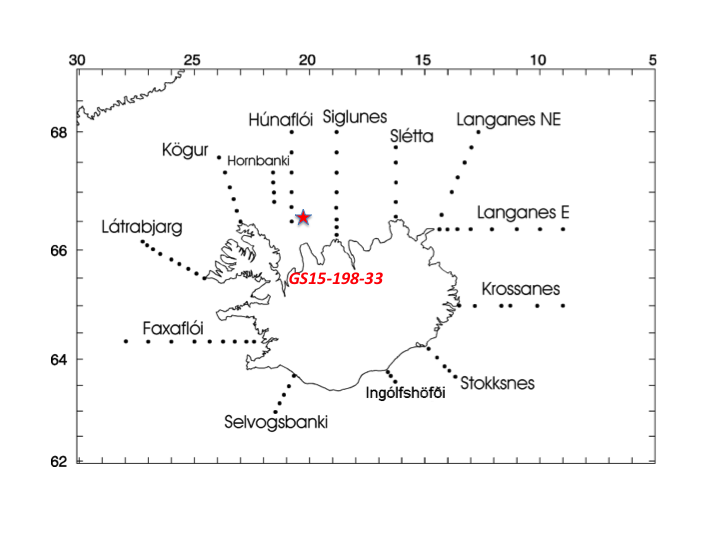


Supplementary Figure: 1


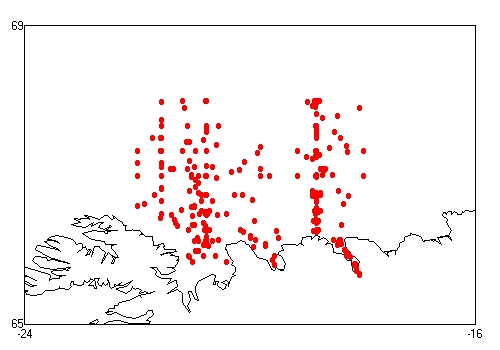


Supplementary Figure: 2

Supplementary Figure: 3

**Supplementary Note 1: Marine sediment core splicing**

Supplementary Figure: 4

**Supplementary Note 2: Chronology**

In this study, we use the age model published in (Perner et al., 2019) that is based on the linear interpolation between anthropogenic time markers. As the core was taken in an open marine environment the applicability of ^210^Pb age-depth models (Appleby 2001; Sanchez-Cabeza and Ruiz Fernandez, 2012; Mabit et al., 2014) is limited. Given the high sedimentation rate it is very likely that lateral matter influx (including ^210^Pb) dominates over atmospheric fallout deposition (this is also evident from the Hg levels in the upper core section, see below and S5 Fig.). This, for example, limits the application of the CRS (Constant Rate of Supply) model (e.g. Appleby 2001; Sanchez-Cabeza and Ruiz Fernandez 2012; Mabit et al. 2014). In S5 Fig. (modified after Perner et al., 2019) the results of a CFCS (Constant Flux and Constant Sedimentation) modelling exercise is shown. The ^210^Pb_unsupp_ shows an exponential decrease with depth which indicates a rather linear sedimentation rate (monotone sedimentation). The CFCS modelling was performed on a depth scale and not on a cumulative mass scale as dry bulk density was unfortunately not measured. Therefore, one basic assumption is that compaction is not severe which is likely the case here. In an open marine environment, there is a need to use artificial time markers to support ^210^Pb (e.g. Smith, 2001; Appleby 2008). Perner et al. (2019) used as additional time markers (S5 Fig.): artificial radionuclides ^137^Cs/^241^Am and pollution derived mercury (Hg), as follows:

*Pollution-derived Hg increase from c. AD 1900*: The assignment of this increase to approx. AD 1900 follows the conclusion of Engstrom et al. (2014) who present an extensive compilation of lake-sediment records of Hg accumulation from remote regions which demonstrate that in the vast majority of lakes Hg started to increase from c. AD 1900. The core depth from which Hg increases above natural background levels marks also an equivalent ^210^Pb dating horizon. The ^210^Pb_unsupp._ based CFCS age model provides an age of AD 1896 +- 15 years for 125 cm core depth.

*Artificial radionuclides ^137^Cs/^241^Am:* Atmospheric bomb testing, starting in 1954, introduced the artificial radionuclides ^137^Cs and ^241^Am into the marine environment and provides a distinctive chronostratigraphic marker. The testing maximum was reached in 1964 and the maximum is also used as a stratigraphic marker.

*Year of sampling, AD 2015:* The sediment surface was assigned to AD 2015 the year of sampling. Clearly, the terrigenous sediment fraction in this fine fraction dominated deposit contains material that is older and laterally transported to give the extremely high sedimentation rates, as also indicated by the relatively high Hg values and rather high ^137^Cs levels in the upper core section. However, given the high sedimentation rate and the high quality of the coring equipment (multi-corer with well-preserved surface sediment characteristics) used it is likely that the foraminifers (proxy) buried by the laterally transported fine-grained terrigenous sediment fraction are modern i.e. AD 2015.

Supplementary Figure: 5

**Supplementary Note 3: Disequilibrium in δ^13^ C of planktic foraminifera**

The δ^13^C_DIC_ in seawater is affected by several processes, however, nutrient cycling and air-sea CO_2_ exchange are the most important ones (Broecker and Maier-Reimer, 1992; Broecker and Peng, 1982). The δ^13^C of planktonic foraminiferal species is often used as a proxy for the δ^13^C_DIC_, which in turn depends on the carbon exchange between atmosphere and ocean, and biological production/respiration.

There is an overall offset of -0.8‰ between the core top *N. pachyderma* δ^13^C_calcite_ measurements (mean value -0.04‰) and the actual mixed layer δ^13^_DIC_ values measured at the estimated calcification depth [~110 m, this study] in July 2015 (Fig. 2). This offset is very similar to that reported for *N. pachyderma* in the north north-west North Atlantic (Jonkers et al., 2013). The offset from ambient water δ^13^_DIC_ can arise from various biological, physical, and chemical processes (Bauch et al., 2002). The most important vital effects influencing the foraminifera itself are (i) ontogenetic/metabolic effects, (ii) photosynthetic activity by symbionts, (iii) temperature, and (iv) carbonate system parameters.

δ^13^C dependency on chemo-physical factors can be summarised in the following 4 points, where points 1 and 2 can affect the δ^13^C_DIC_ whereas point 3 and 4 affect the δ^13^C_foram_:

1. Phosphate and δ^13^C have an inverse relationship, hence low-nutrient, low phosphate water masses have a high δ^13^C signature and vice versa. The δ^13^C of oceanic carbon increases by 1.1‰ for every 1 µmol kg^-1^ decrease in oceanic PO_4_ (Broecker and Maier-Reimer, 1992). Primary productivity processes fractionate the surface/subsurface water mass towards more positive δ^13^C values as the lighter ^12^C is preferentially incorporated during photosynthesis.
2. The Marine Suess effect is a negative δ^13^C shift brought about by the ocean uptake of isotopically light carbon (δ^13^C of atmospheric CO_2_) from the burning of fossil fuels (Eide et al., 2017).
3. Carbon isotope fractionation is temperature dependent with a positive δ^13^C shift under cooling with a slope of -0.17 ‰/°C; (Jonkers et al., 2013).
4. δ^13^C values depend on the carbonate chemistry of seawater with a decrease in foraminifera δ^13^C under increasing CO_3_^-2^ concentration of the surrounding seawater (Spero et al., 1997; Zeebe, 1999).

Besides, there is a difference in the δ^13^C_DIC_ ratio of SPMW and AIW of ~0.6‰, with typical modern SPMW and AIW δ^13^C DIC values of ~1.4‰ and 2.0‰, respectively (Olsen and Ninnemann, 2010; Tagliabue and Bopp, 2008). Shifts in the relative proportion of SPMW and AIW entrained onto the North Icelandic shelf could therefore lead to changes in δ^13^C DIC. Upwelling processes can bring deeper ^12^C-enriched carbon water masses up to the surface related to wind forcing changes. At the core location, AIWs have a more negative signature than their overlaying SPMW counterparts (Fig. 2).


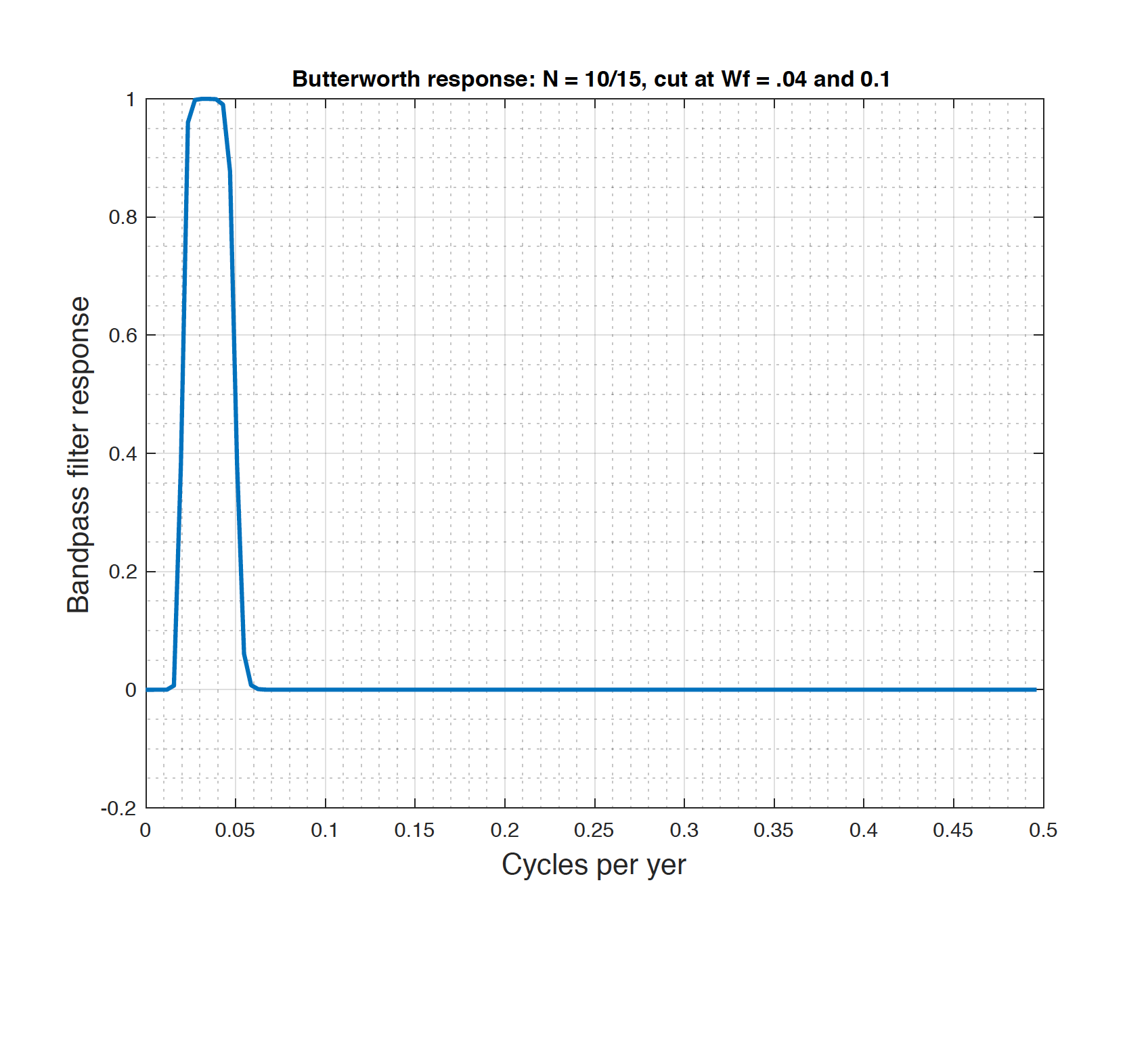


Supplementary Figure: 6

Supplementary Figure: 7

Supplementary Figure: 8

Supplementary Figure: 9


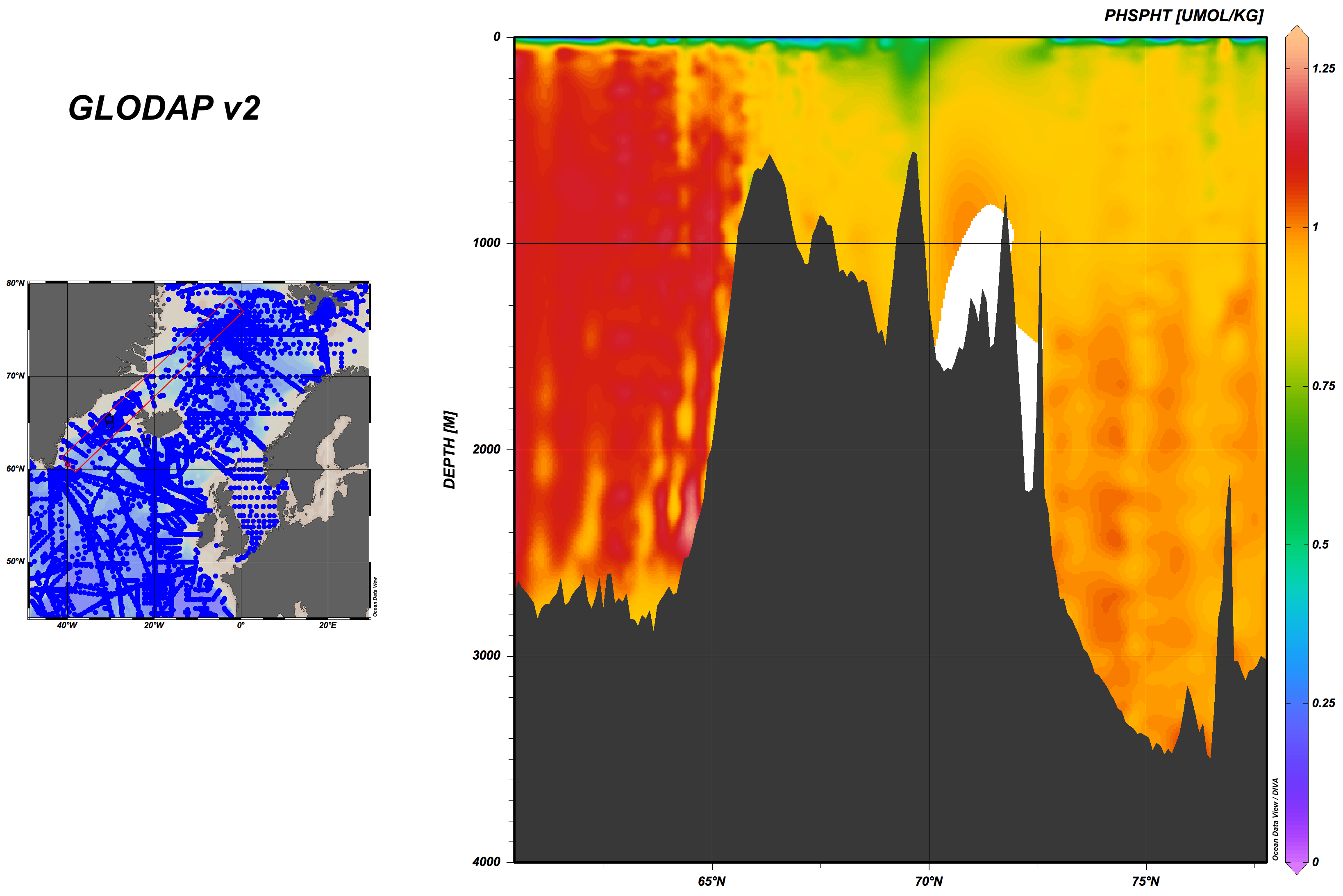


Supplementary Figure: 10

Supplementary Figure: 11


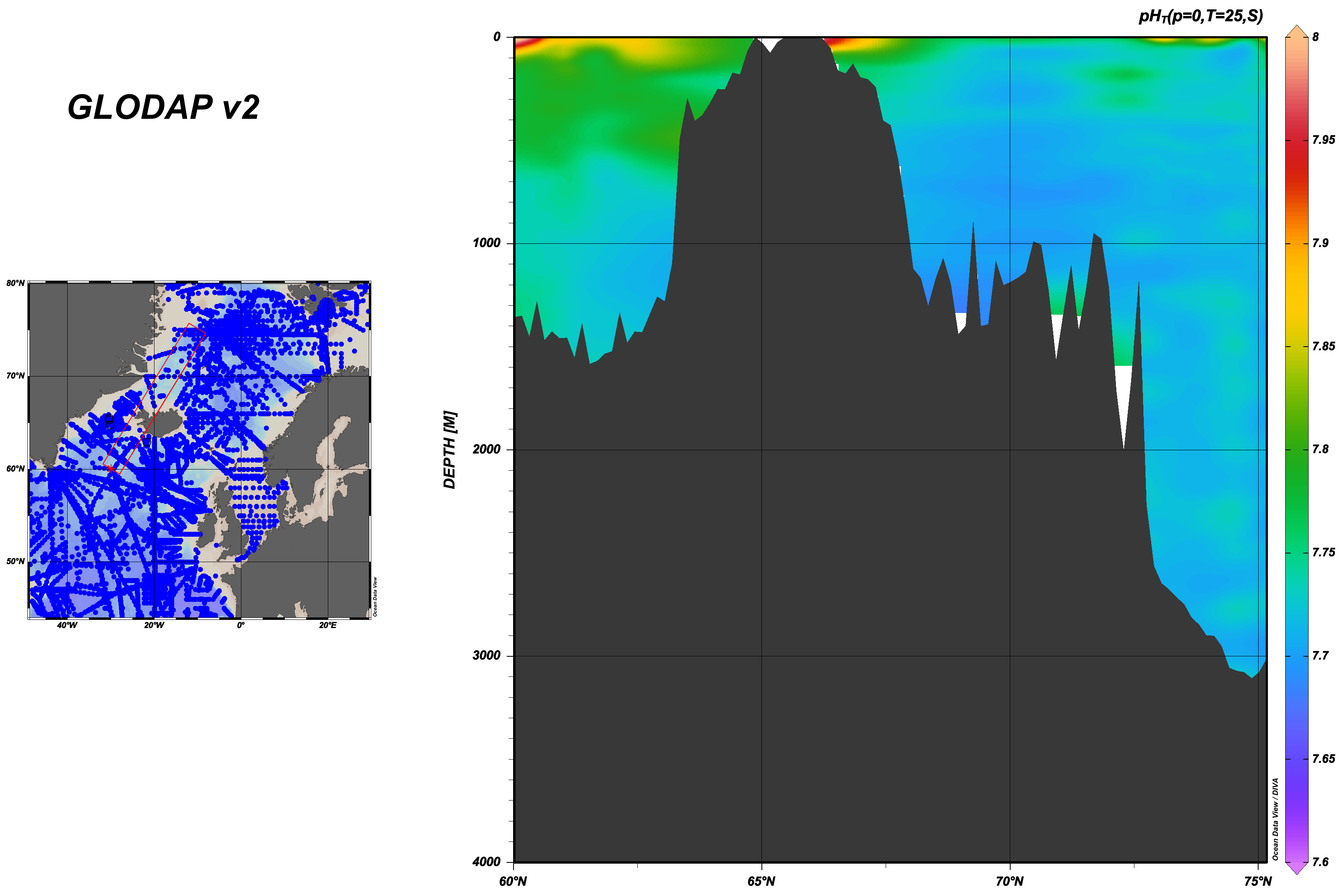


Supplementary Figure: 12

**Supplementary References**

Appleby, P.G., 2008. Three decades of dating recent sediments by fallout radionuclides: a review. The Holocene 18, 83-93.

Appleby P. G. (2001) Chronostratigraphic techniques in recent sediments. In Tracking Environmental Change Using Lake Sediments, vol. 1 (eds. W. M. Last and J. P. Smol), pp. 171–201. Basin Analysis, Coring and Chronological Techniques. Kluwer Academic Publishers, Dordrecht, The Netherlands.

Engstrom, D. R., Fitzgerald, W. F., Cooke, C. A., Lamborg, C. H., Drevnick, P. E., Swain, E. B., Balogh, S. J., & Balcom, P. H. (2014). Atmospheric Hg emissions from preindustrial gold and silver extraction in the Americas: A reevaluation from lake-sediment archives. *Environmental science & technology*, *48*(12), 6533-6543.

Mabit, L., Benmansour, M., Abril, J. M., Walling, D. E., Meusburger, K., Iurian, A. R., Bernard, C.,

Tarjan, S., Owens, P. N., Blake, W. H. & Alewell, C. (2014). Fallout 210Pb as a soil and sediment tracer in catchment sediment budget investigations: a review. *Earth-Science Reviews*, *138*, 335-351.

Perner, K., Moros, M., Otterå, O.H., Blanz, T., Schneider, R.R., Jansen, E., 2019. An oceanic perspective on Greenland’s recent freshwater discharge since 1850. Scientific Reports 9, 17680.

Sanchez-Cabeza, J. A., & Ruiz-Fernández, A. C. (2012). 210Pb sediment radiochronology: an integrated formulation and classification of dating models. *Geochimica et Cosmochimica Acta*, *82*, 183-200.

Smith, J.N., 2001. Why should we believe 210Pb sediment geochronologies? Journal of Environmental Radioactivity 55, 121-123.

Bauch, D., Erlenkeuser, H., Winckler, G., Pavlova, G., Thiede, J., 2002. Carbon isotopes and habitat of polar planktic foraminifera in the Okhotsk Sea: the ‘carbonate ion effect’ under natural conditions. Marine Micropaleontology, 45(2): 83-99.

Broecker, W.S., Maier-Reimer, E., 1992. The influence of air and sea exchange on the carbon isotope distribution in the sea. Global Biogeochemical Cycles, 6(3): 315-320.

Broecker, W.S., Peng, T.-H., 1982. Tracers in the Sea. Lamont-Doherty Geol. Obs, Columbia Univ., Palisades, NY p. 690a.

Eide, M., Olsen, A., Ninnemann, U.S., Eldevik, T., 2017. A global estimate of the full oceanic 13C Suess effect since the preindustrial. Global Biogeochemical Cycles, 31(3): 492-514.

Jonkers, L., Heuven, S., Zahn, R., Peeters, F.J.C., 2013. Seasonal patterns of shell flux, δ18O and δ13C of small and large N. pachyderma (s) and G. bulloides in the subpolar North Atlantic. Paleoceanography, 28(1): 164-174.

Olsen, A., Ninnemann, U., 2010. Large δ13C gradients in the Preindustrial North Atlantic Revealed. Science, 330(6004): 658-659.

Perner, K. et al., 2019. An oceanic perspective on Greenland’s recent freshwater discharge since 1850. Scientific Reports, 9(1): 17680.

Spero, H.J., Bijma, J., Lea, D.W., Bemis, B.E., 1997. Effect of seawater carbonate concentration on foraminiferal carbon and oxygen isotopes. Nature, 390: 497.

Tagliabue, A., Bopp, L., 2008. Towards understanding global variability in ocean carbon-13. Global Biogeochemical Cycles, 22(1).

Zeebe, R.E., 1999. An explanation of the effect of seawater carbonate concentration on foraminiferal oxygen isotopes. Geochimica et Cosmochimica Acta, 63(13): 2001-2007.
